# Altered Intra-Nuclear Organisation of Heterochromatin and Genes in ICF Syndrome

**DOI:** 10.1371/journal.pone.0011364

**Published:** 2010-06-29

**Authors:** Andrew Jefferson, Stefano Colella, Daniela Moralli, Natalie Wilson, Mohammed Yusuf, Giorgio Gimelli, Jiannis Ragoussis, Emanuela V. Volpi

**Affiliations:** 1 Wellcome Trust Centre for Human Genetics, University of Oxford, Oxford, United Kingdom; 2 Laboratorio di Citogenetica, Istituto G. Gaslini, Genova, Italy; Duke University, United States of America

## Abstract

The ICF syndrome is a rare autosomal recessive disorder, the most common symptoms of which are immunodeficiency, facial anomalies and cytogenetic defects involving decondensation and instability of chromosome 1, 9 and 16 centromeric regions. ICF is also characterised by significant hypomethylation of the classical satellite DNA, the major constituent of the juxtacentromeric heterochromatin. Here we report the first attempt at analysing some of the defining genetic and epigenetic changes of this syndrome from a nuclear architecture perspective. In particular, we have compared in ICF (Type 1 and Type 2) and controls the large-scale organisation of chromosome 1 and 16 juxtacentromeric heterochromatic regions, their intra-nuclear positioning, and co-localisation with five specific genes (*BTG2*, *CNN3*, *ID3*, *RGS1*, *F13A1*), on which we have concurrently conducted expression and methylation analysis. Our investigations, carried out by a combination of molecular and cytological techniques, demonstrate the existence of specific and quantifiable differences in the genomic and nuclear organisation of the juxtacentromeric heterochromatin in ICF. DNA hypomethylation, previously reported to correlate with the decondensation of centromeric regions in metaphase described in these patients, appears also to correlate with the heterochromatin spatial configuration in interphase. Finally, our findings on the relative positioning of hypomethylated satellite sequences and abnormally expressed genes suggest a connection between disruption of long-range gene-heterochromatin associations and some of the changes in gene expression in ICF. Beyond its relevance to the ICF syndrome, by addressing fundamental principles of chromosome functional organisation within the cell nucleus, this work aims to contribute to the current debate on the epigenetic impact of nuclear architecture in development and disease.

## Introduction

The Immunodeficiency, Centromeric region instability and Facial anomalies (ICF) syndrome (OMIM 242860) is a rare autosomal recessive disorder often fatal in childhood [1]. So far, less than 50 cases have been reported worldwide.

The ICF syndrome is characterised by phenotypic and clinical variability, with the most consistent features being reduction in serum immunoglobulin (Ig) levels, developmental delay, facial anomalies and cytogenetic defects. The normal cause of death in ICF patients is infection, usually of the pulmonary or gastrointestinal tract [rev. in [2]].

Cytogenetic defects of diagnostic significance principally involve decondensation of the juxtacentromeric (or centromere adjacent) heterochromatic regions of chromosomes 1 and 16, and to a lesser extent chromosome 9. In mitogen-stimulated lymphocytes, a wide array of aberrations can be observed, ranging from greatly stretched heterochromatic regions to multiradiate chromosomes. The juxtacentromeric heterochromatic regions of chromosome 1 and 16 are mainly comprised of classical satellite 2 and 3 repeats. Chromosome fusion in the ICF syndrome occurs only at regions of decondensed centromere-adjacent heterochromatin, and the alpha satellite repeats, the main component of centromeres, always remain outside the regions of multiradiate chromosome fusions [3], [4]. Lymphoblastoid cell lines generated from ICF patients also show high frequencies of the same karyotypic abnormalities as those observed in mitogen-stimulated lymphocytes [5], [6].

ICF syndrome is also characterised by abnormal DNA methylation. Although only a slight decrease in 5-methylcytosine has been observed at the overall genomic level [7], the classical satellite 2 DNA sequences are significantly and consistently hypomethylated at cytosine residues in this syndrome [3], [8], [9], [10]. Chromosome 9 juxtacentromeric heterochromatin, which mainly consists of related satellite 3 DNA is also hypomethylated, although to a lesser extent [9], [11]. A small number of other genomic regions show significant hypomethylation in ICF syndrome, most notably the non-satellite repeats D4Z4 and *NBL2* [10]. Single copy loci showing heterogeneous hypomethylation comprise *SCP-1* [12], the imprinted loci D15S9, D15S63 and H19 [3] and in female ICF cells a number of genes residing on the inactive X chromosome [3], [12], [13]. Also, some significant changes in DNA methylation patterns at promoters and CpG rich regions were recently identified within a sample of dysregulated genes in ICF [14].

ICF syndrome was initially linked to chromosome 20q11.2 [15] and subsequently the DNA methyltransferase 3B gene (*DNMT3B*) was identified as the gene responsible for the methylation defects observed in ICF [7]. Along with DNMT3A, DNMT3B acts to methylate cytosine residues *de novo* and is essential for normal development [16].

Mutations of *DNMT3B* in ICF syndrome are heterogeneous. Analysis of fourteen patients revealed eleven different mutations, including eight different missense mutations, two nonsense mutations and a splice site mutation [17]. Nonsense mutations always occur as compound heterozygous, highlighting that the DNMT3B protein is essential for life. Most recently, a model for ICF syndrome has been engineered by generating *Dnmt3b* mutations in mice [18]. Homozygous mice carrying two missense alleles of *Dnmt3b* show many ICF-like characteristics, including hypomethylation of heterochromatin repeat DNA.

Wijmenga and collaborators [17] also identified five ICF patients who do not carry mutations in the *DNMT3B* gene. More recent investigations described further patients who did not carry a mutation of *DNMT3B* [19], [20]. Intriguingly, Jiang and co-authors showed that the subset of patients carrying a mutation in the *DNMT3B* gene had alpha satellite methylation patterns comparable to control samples. In contrast, the subset of patients who did not carry mutations in *DNMT3B* exhibited hypomethylation of the alpha satellite as well as classical satellite DNA. These findings lead to the proposal of the existence of two distinct types of ICF syndrome, namely a Type 1, in which patients display mutations in the *DNMT3B* gene, but have normal alpha satellite methylation, and a Type 2, characterised by normal *DNMT3B* and hypomethylation of alpha satellite DNA [19].

Global expression studies by microarray analysis have identified significant changes in the expression of several hundreds of genes in ICF, involved in immune function, development and neurogenesis as well as lymphogenesis, signal transduction and apoptosis [14], [21].

Over the years, several hypotheses linking altered gene expression to the hypomethylation of juxtacentromeric heterochromatin in ICF have been postulated by different research groups, commonly suggesting inappropriate release or recruitment of regulatory complexes by the hypomethylated satellite DNA, affecting the regulatory properties of the heterochromatin [7], [8], [13], [21], [22].

These suggestions have prompted us to investigate whether the decondensation of the juxtacentromeric heterochromatin, as observed in metaphase, and general chromosomal instability reported in ICF patients, correspond to changes in the three-dimensional properties of the heterochromatin in interphase; our working hypothesis being that disruption to the heterochromatin spatial configuration may interfere with transcriptional silencing and be indirectly responsible for some of the changes in gene expression accounting for the symptoms of ICF.

Accordingly, we have analysed and compared in two patients (ICF Type 1 and ICF Type 2) and both related (unaffected parents) and unrelated controls the large-scale organisation and intra-nuclear positioning of chromosome 1 and 16 juxtacentromeric heterochromatin. Heterochromatin organisation and positioning have been analysed in different cell lines and cultures, including, as well as ICF cells presenting different degrees of classical satellite 2 hypomethylation [8], control cells in which DNA hypomethylation had been experimentally induced by treatment with 5-azacytidine. We have also carried out a comparative quantification of chromosome 1 satellite 2 and 3 repeats in ICF cells and controls. Finally, we have analysed and compared the intra-nuclear positioning of four genes from chromosome 1 and one gene from chromosome 6 – namely *BTG2* (B-cell translocation gene 2) (1q32), *CNN3* (Calponin 3) (1p22-p21), *ID3* (Inhibitor of DNA binding 3)(1p36.13-p36.12), *RGS1* (Regulator of G protein signalling) (1q31) and *F13A1* (Factor XIII; A1 subunit) (6p25-p24) - and their co-localisation with the juxtacentromeric heterochromatin of chromosome 1. The expression of these genes had previously been reported to be altered in ICF [21]. We have assessed and compared their expression levels in our patients and control cell lines, and, for three of them, we have also analysed in detail the methylation status of upstream CpG islands of their promoters using a quantitative methylation assay.

Beyond its relevance to the ICF syndrome, by addressing fundamental principles of chromosome functional organisation within the cell nucleus, this work aims to contribute to the current debate on the epigenetic impact of nuclear architecture in development and disease.

## Results

### The large-scale organisation of chromosome 1 juxtacentromeric heterochromatin is altered in ICF B-cells

**Figure 1:**
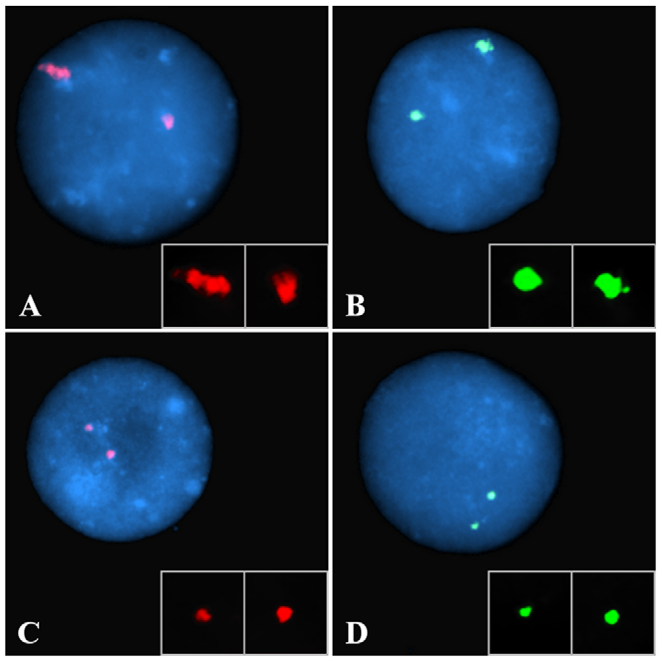
Visualisation of the juxtacentromeric heterochromatin in the cell nucleus. Chromosome 1 and chromosome 16 juxtacentromeric heterochromatic regions were visualised by hybridising to interphase nuclei (counterstained with DAPI) (blue) the classical satellite DNA probes D1Z1 (red) and D16Z3 (green), respectively. Evaluation of the hybridisation signals on a cell-by-cell basis allowed the identification of a pronounced inter-nuclear and inter-allelic variability in the heterochromatin patterns within each of the cell populations (either patients or controls). Above are examples of two easily distinguishable configurations: “conventional” (the fluorescent signal is typically conspicuous and its outline is uneven) (A and B) and “compact” (the fluorescent signal is conspicuously smaller and its outline well defined) (C and D). Single channel images were imaged using a monochrome CCD camera attached to a wide-field fluorescence microscope, pseudocoloured and merged. Insert boxes within each image show the heterochromatic signal digitally enhanced.

Observations on the heterochromatin in interphase, conducted in parallel by wide-field and confocal microscopy, revealed some significant differences between ICF cells and controls. Chromosome 1 juxtacentromeric heterochromatin, as defined by hybridisation with the classical satellite DNA probe D1Z1, and chromosome 16 juxtacentromeric heterochromatin, as defined by hybridisation with the classical satellite DNA probe D16Z3, were analysed and compared in B-lymphoblastoid cell nuclei of two different patients (ICF Patient 1 presenting with ICF Type 1 and ICF Patient 2 presenting with ICF Type 2) and three controls, two of which were unaffected parents of the ICF patients (respectively called Control 1 and Control 2) and one a normal unrelated B-lymphoblastoid cell line (DO208915). Evaluation of the fluorescent hybridisation signals on a per cell basis on 2D fixed cells (2D FISH) allowed us to identify in each cell population the co-existence of noticeably different hybridisation patterns (Examples in Fig. 1).

**Figure 2:**
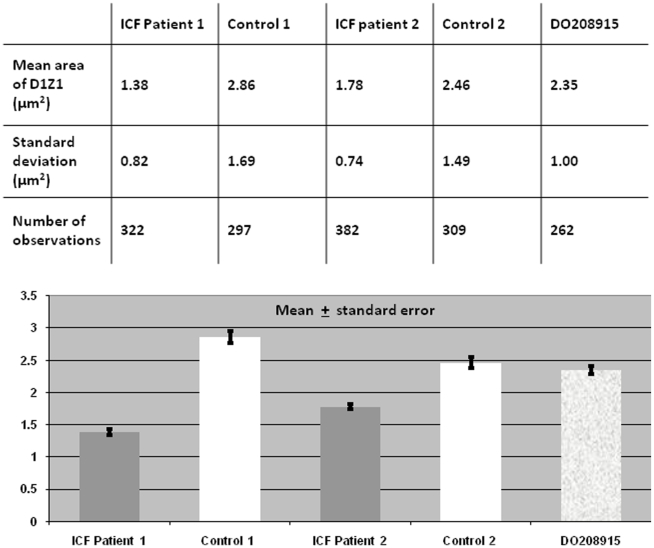
Chromosome 1 juxtacentromeric heterochromatin: area measurements in ICF cells and controls. Measurements of the areas occupied by chromosome 1 juxtacentromeric heterochromatin, as defined by hybridisation with the D1Z1 classical satellite DNA probe, were carried out in hundreds of 2D-fixed interphase nuclei for each cell line and the mean values calculated. The measurements were performed as described in the Materials and Methods. The data sets were compared using a Kolmogorov-Smirnov test, which revealed a statistically significant difference between ICF patients and Controls altogether (D = 0.3480, P<0.001).

In order to quantify possible cumulative differences in the large-scale organisation of the heterochromatin of chromosome 1 in ICF B-cells and controls, we measured the intra-nuclear areas occupied by the juxtacentromeric heterochromatic regions, as defined by hybridisation on 2D-fixed interphase nuclei with the corresponding classical satellite DNA probe (Fig. 2). The measurements were performed as described in the Materials and Methods. The data sets were compared using a Kolmogorov-Smirnov test, which revealed a statistically significant difference between ICF and Control cells (D = 0.3480, P<0.001).

**Figure 3:**
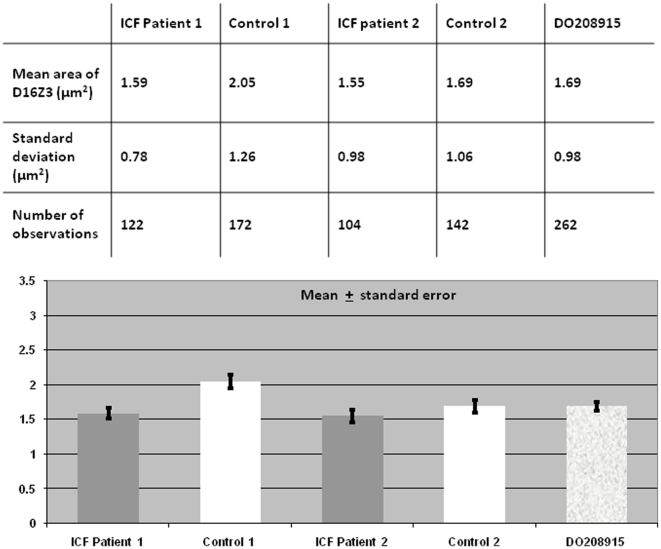
Chromosome 16 juxtacentromeric heterochromatin: area measurements in ICF cells and controls. Measurements of the areas occupied by chromosome 16 juxtacentromeric heterochromatin, as defined by hybridisation with the D16Z3 classical satellite DNA probe, were obtained in hundreds of 2D-fixed interphase nuclei per cell line and the mean values calculated. The measurements were performed as described in the Materials and Methods. The data sets were compared using a Kolmogorov-Smirnov test and no statistically significant difference was observed between ICF patients and Controls altogether (D = 0.1029, P = 0.076).

Similar to that observed for chromosome 1, when measurements of the hybridisation areas of chromosome 16 juxtacentromeric heterochromatin were obtained and averaged, this heterochromatic region appeared to be smaller in ICF nuclei compared to controls (Fig. 3). However, when the data sets were compared, no statistically significant difference was observed between ICF and Control (D = 0.1029, P = 0.076).

**Figure 4:**
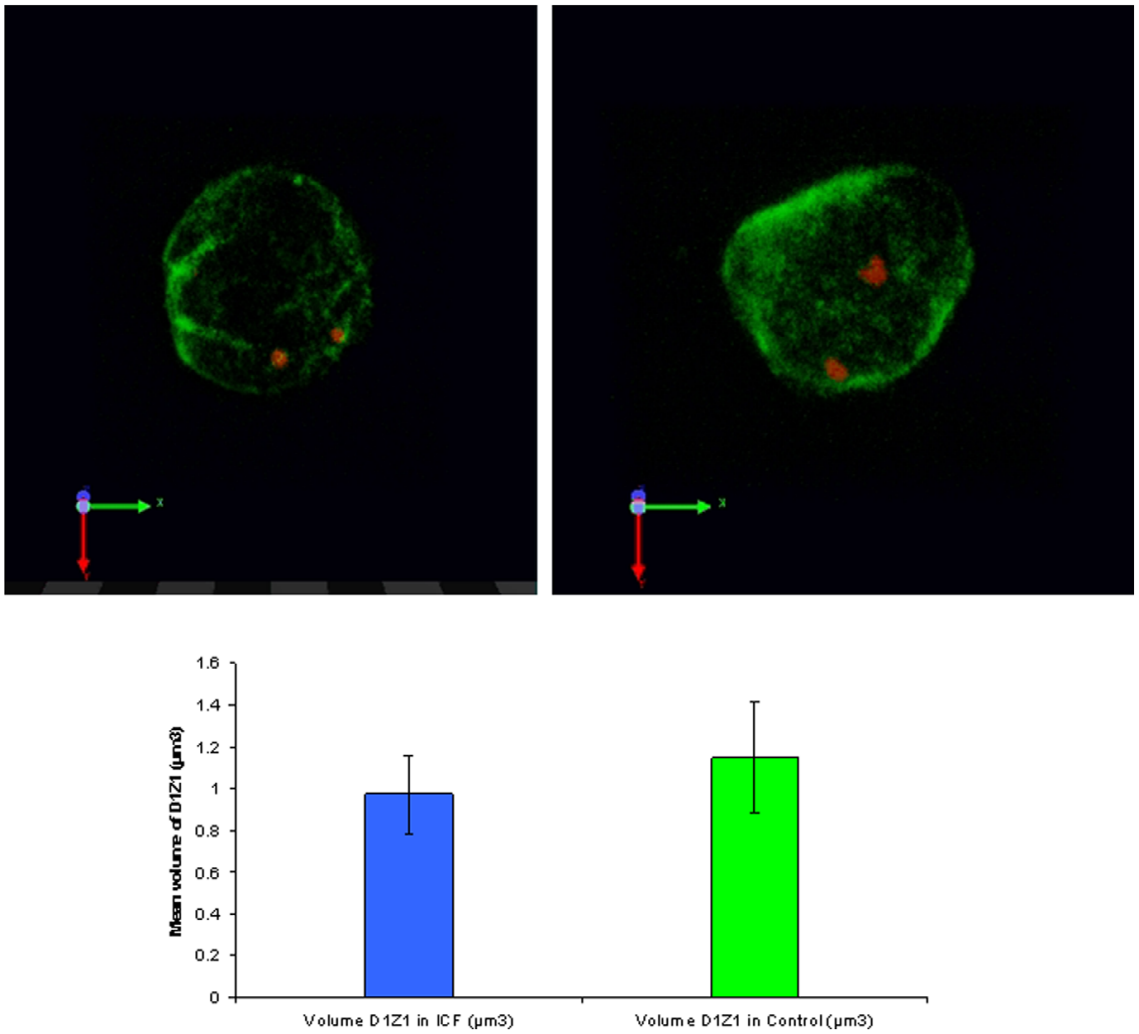
Chromosome 1 juxtacentromeric heterochromatin: volume measurements in ICF cells and controls. The nuclear volumes occupied by the chromosome 1 juxtacentromeric heterochromatin, as defined by hybridisation with the D1Z1 classical satellite DNA probe (red) on 3D-fixed interphase nuclei (immunostained with anti-Lamin B, green), were measured and compared in ICF Patient 1 and Control 1 as described in the Materials and Methods. The heterochromatin was shown to occupy on average a smaller volume in ICF cells. The data sets were compared using a Kolmogorov-Smirnov test, which revealed a statistically significant difference between the ICF and Control volume distributions (D = 0.2356, P<0.001). Examples of variable heterochromatin patterns as observed by 3D-FISH, confocal analysis and volume reconstruction are shown in the top panels.

To further corroborate the results obtained by 2D FISH, the large-scale organisation of chromosome 1 juxtacentromeric heterochromatin was also investigated and compared in ICF Patient 1 and Control 1 by means of 3D FISH, a variant of the hybridisation technique believed to better preserve nuclear architecture, followed by laser scanning confocal microscopy analysis (Fig. 4). Volume measurements were carried out as described in the Materials and Methods. The heterochromatin was shown to occupy on average a smaller volume in ICF cells, with a mean value of 0.970 µm^3^ (SD = 0.37) (N = 202), whilst in the Control the mean value was 1.147 µm^3^ (SD = 0.53) (N = 147). The data sets were compared using a Kolmogorov-Smirnov test, which revealed a statistically significant difference between the ICF and Control volume distributions (D = 0.2356, P<0.001).

Contrary to the consensual view that decondensation and stretching of the juxtacentromeric heterochromatic regions, as generally observed in metaphase in ICF, should be expected to correspond to decondensation and stretching in interphase, our findings on the large-scale organisation of these chromosomal land-marks in B-cells show that the ICF nuclear phenotype – when compared to controls - is characterised by an apparently more compact spatial configuration of the juxtacentromeric heterochromatin.

### Variability of chromosome 1 juxtacentromeric heterochromatin organisation in interphase is not cell-cycle related

In order to establish whether the variability in the configuration of chromosome 1 juxtacentromeric heterochromatin, observed between and within cell cultures, could be related to dissimilarities in the rate of proliferation and progression through the cell-cycle between ICF cells and controls, we conducted some comparative tests to assess the existence of a temporal connection between the different heterochromatin patterns and specific stages of the cell-cycle.

First, we investigated possible cell cycle stage composition differences between ICF and control cell lines by FACS analysis (Figure S1).These investigations revealed very similar percentages of diploid cells in G1, S and G2 for the ICF Patient 1 and Control 1, and for the ICF Patient 2 and Control 2 samples analysed, ruling out significant differences in cell cycle stage composition between the patient and control cell lines.

**Figure 5:**
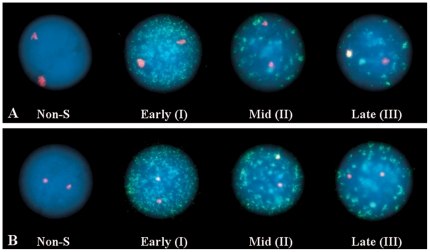
Heterochromatin configuration at different stages of the cell-cycle. BrdU pulse labelling (green), to visualise cells undergoing DNA synthesis (S phase), was used in conjunction with FISH with the D1Z1 probe (red) to identify a possible connection between the observed variability in the configuration of chromosome 1 juxtacentromeric heterochromatin and progression through the cell-cycle. Comparative tests were conducted in both ICF and controls. No differences were observed in the heterochromatin organisation when comparing B-cell nuclei in both ICF and control cells, with “conventional” (A) and “compact” (B) hybridisation patterns equally present in non-S phase and the various stages of the S phase of the cell-cycle. Examples above belong to a miscellanea of informative pictures collected from both ICF and control cells. S phase progression patterns as described in O'Keefe [23].

Then, we hybridised chromosome 1 classical satellite probe D1Z1 to BrdU pulse-labelled B-lymphoblastoid cells from ICF Patient 1 and Control 1 (Fig. 5). Cells undergoing DNA replication (S-phase) were visualised by antibody detection of incorporated BrdU. The different immunolabelling patterns were interpreted according to O'Keefe et al. [23]. The “conventional“ (Fig. 5A) and “compact” (Fig. 5B) heterochromatin patterns appeared to be present indiscriminately during S phase progression and non-S phase of the cell cycle in both Control 1 (N = 91) and ICF Patient 1 (N = 70).

Based on both our FACS analysis and our BrdU incorporation experiments, we conclude that proliferation status and progression through cell-cycle can be excluded as factors responsible for the variability in the spatial configuration of juxtacentromeric heterochromatin observed between and within cell populations.

### The intra-nuclear positioning of chromosome 1 juxtacentromeric heterochromatin is also altered in ICF B-cells

**Figure 6:**
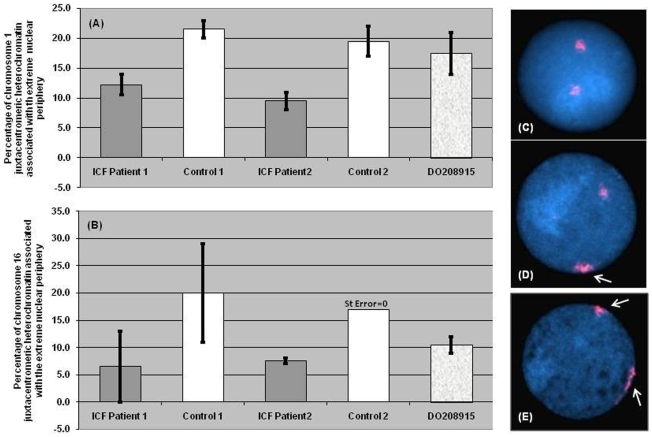
Association of chromosome 1 and 16 juxtacentromeric heterochromatin with the extreme nuclear periphery. Preferential positioning of the chromosome 1 (A) and chromosome 16 (B) juxtacentromeric heterochromatin at the nuclear periphery was assessed in ICF Patient 1 and 2, Control 1 and 2, and DO208915. The heterochromatic signals were considered to be positioned at the extreme nuclear periphery if any part of them appeared to associate with the nuclear rim, as defined by the edge of the DAPI staining. Panels C, D and E respectively show examples of nuclei where none or either one or both chromosome 1 heterochromatic areas (D1Z1 signal in red) map at the extreme nuclear periphery (as indicated by the white arrows). The reduction in association of the D1Z1 and D16Z3 hybridisation signals with the extreme nuclear periphery in ICF B-cell lines compared to controls is in both cases statistically significant using a Chi-squared goodness of fit test, respectively (χ^2^ = 4.882, P = 0.027) and (χ^2^ = 10.563, P = 0.001).

The positioning of chromosomes 1 and 16 juxtacentromeric heterochromatin in interphase was also assessed and compared in B-lymphoblastoid cells from ICF patients and controls. Preliminary observations were aimed at establishing whether chromosome 1 and chromosome 16 centromeric regions showed preferential association with the extreme nuclear periphery, and identifying possible differences between patients and controls. Preferential positioning of chromosome 1 juxtacentromeric heterochromatin at the nuclear periphery was assessed in ICF Patient 1 and 2, Control 1 and 2, and DO208915.The heterochromatic signal was considered to be positioned at the extreme nuclear periphery when any part of it appeared to associate with the nuclear rim, as defined by the edge of the DAPI staining (Examples in Fig. 6). These observations were conducted independently from any consideration on the heterochromatin configuration, and a minimum of 100 nuclei for each experiment were scored randomly.

Our observations on control B-cells showed no marked preferential positioning at the extreme nuclear periphery for chromosome 1 juxtacentromeric heterochromatin, with less than 25% of D1Z1 signals normally associating with the nuclear rim in all cell lines analysed (Fig. 6A). Interestingly, in ICF B-cell nuclei, juxtacentromeric heterochromatin positioning at the extreme nuclear periphery was less frequent, with the percentage of D1Z1 signals associating with the nuclear rim being less than 15% in all cells cultures analysed. The reduction in association of the D1Z1 hybridisation signal with the extreme nuclear periphery in ICF B-cell lines compared to controls is statistically significant using a Chi-squared goodness of fit test (χ^2^ = 4.882, P = 0.027).

Similar observations on the intranuclear positioning of chromosome 16 juxtacentromeric heterochromatin were conducted on ICF Patient 1 and 2, Control 1 and 2, and DO208915 (Fig. 6B). As observed for chromosome 1, there was no evidence of preferential positioning of chromosome 16 juxtacentromeric heterochromatin at the extreme nuclear periphery in either ICF or control nuclei, but the reduction in association of the heterochromatin with the extreme nuclear periphery observed between ICF and control cell lines proved to be statistically significant (χ^2^ = 10.563, P = 0.001).

**Figure 7:**
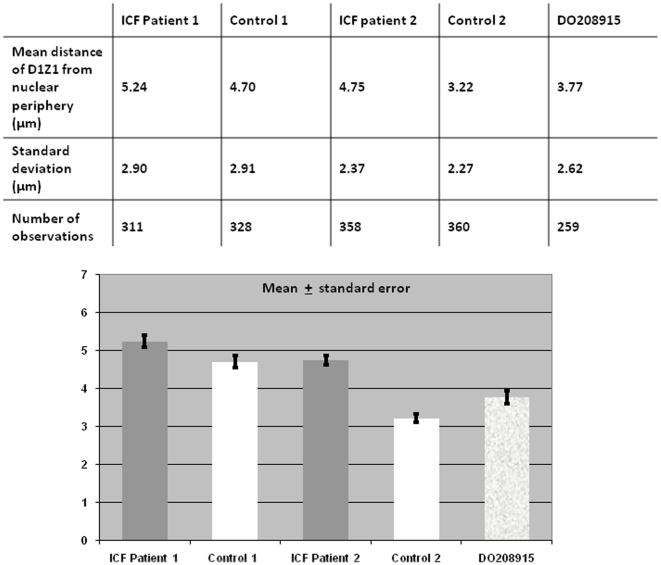
Chromosome 1 juxtacentromeric heterochromatin positioning in relation to the extreme nuclear periphery: distance measurements in ICF cells and controls. The average distance between the centroid of the chromosome 1 juxtacentromeric heterochromatic signal and the extreme nuclear periphery, as defined by the edge of the DAPI nuclear staining, was calculated in unsynchronised B-lymphoblastoid cells from ICF Patients 1 and 2, Controls 1 and 2 and DO208915. When performing a Kolmogorov-Smirnov test, the distance of chromosome 1 juxtacentromeric heterochromatin from the nuclear periphery is significantly greater in ICF cell lines when compared to control cell lines (D = 0.1405, P<0.001).

In order to further evaluate the observed differences in the intra-nuclear positioning between ICF and controls, the distance from the centroid of each juxtacentromeric heterochromatin signal to the nuclear rim was measured as described in Materials and Methods, and averaged in each cell line. To obtain the mean distance of D1Z1 from the extreme nuclear periphery, measurements from two independent experiments were carried out for each cell line (Fig. 7). To take into consideration the varying sizes of nuclei, the measurements were normalised. When performing a Kolmogorov-Smirnov test, the distance of chromosome 1 juxtacentromeric heterochromatin from the nuclear periphery was significantly greater in ICF cell lines when compared to control cell lines (D = 0.1405, P<0.001).

**Figure 8:**
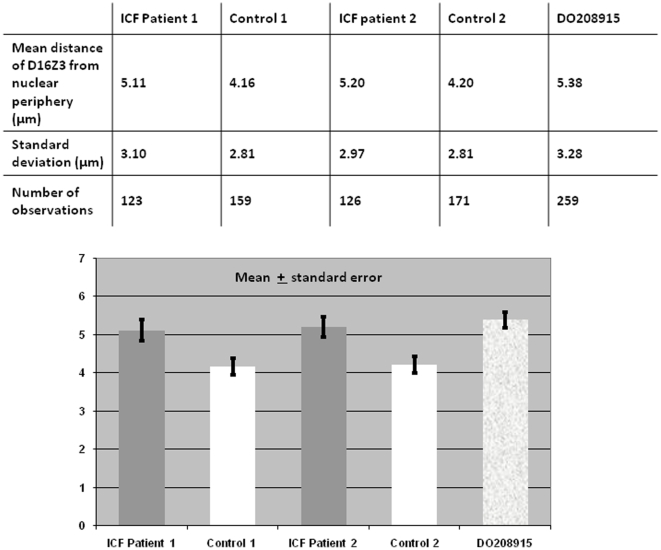
Chromosome 16 juxtacentromeric heterochromatin positioning in relation to the extreme nuclear periphery: distance measurements in ICF cells and controls. The average distance between the centroid of the chromosome 16 juxtacentromeric heterochromatic signal and the extreme nuclear periphery, as defined by the edge of the DAPI nuclear staining, was calculated in unsynchronised B-lymphoblastoid cells from ICF Patients 1 and 2, Controls 1 and 2 and DO208915. In contrast to previous observations, when performing a Kolmogorov-Smirnov test, there is no significant difference in the distance of D16Z3 to the nuclear periphery between ICF and normal cell lines (D = 0.0558, P = 0.678).

The distance of the D16Z3 signal from the extreme nuclear periphery was also measured in B-lymphoblastoid interphase cells from ICF Patient 1 and 2, Control 1 and 2 and DO208915 (Fig. 8). As for chromosome 1, the different sizes of nuclei were taken into consideration by calculating the ratios between distance from nuclear periphery and nuclear radius. However, in contrast to previous observations, there was no significant difference in the distance of D16Z3 to the nuclear periphery between ICF and normal cell lines (D = 0.0558, P = 0.678).

In ICF B-cells, when compared to controls, the degree of association of the hypomethylated chromosome 1 classical satellite DNA signals with the nuclear periphery is lower, suggesting a specific re-positioning of the juxtacentromeric heterochromatin to a more internal location within the nuclear volume.

### Changes in the heterochromatin configuration, as observed in ICF nuclei, can be partially replicated in control cells by treatment with a demethylating agent

It was previously shown that it is possible to reproduce in normal lymphoblastoid cell lines many of the cytological anomalies observed at metaphase in ICF syndrome, in particular the high frequency of juxtacentromeric rearrangements of chromosome 1, by treatment with global demethylating agents, such as 5-azacytidine and 5-azadeoxycytidine [24], [25], [26]. In order to investigate whether treatment with a demethylating agent was also able to reproduce in normal cells the changes to the large-scale organisation and spatial positioning of the heterochromatin observed by us in interphase in this syndrome, we conducted further observations on our cell lines after treatment with 5-azacytidine.

**Figure 9:**
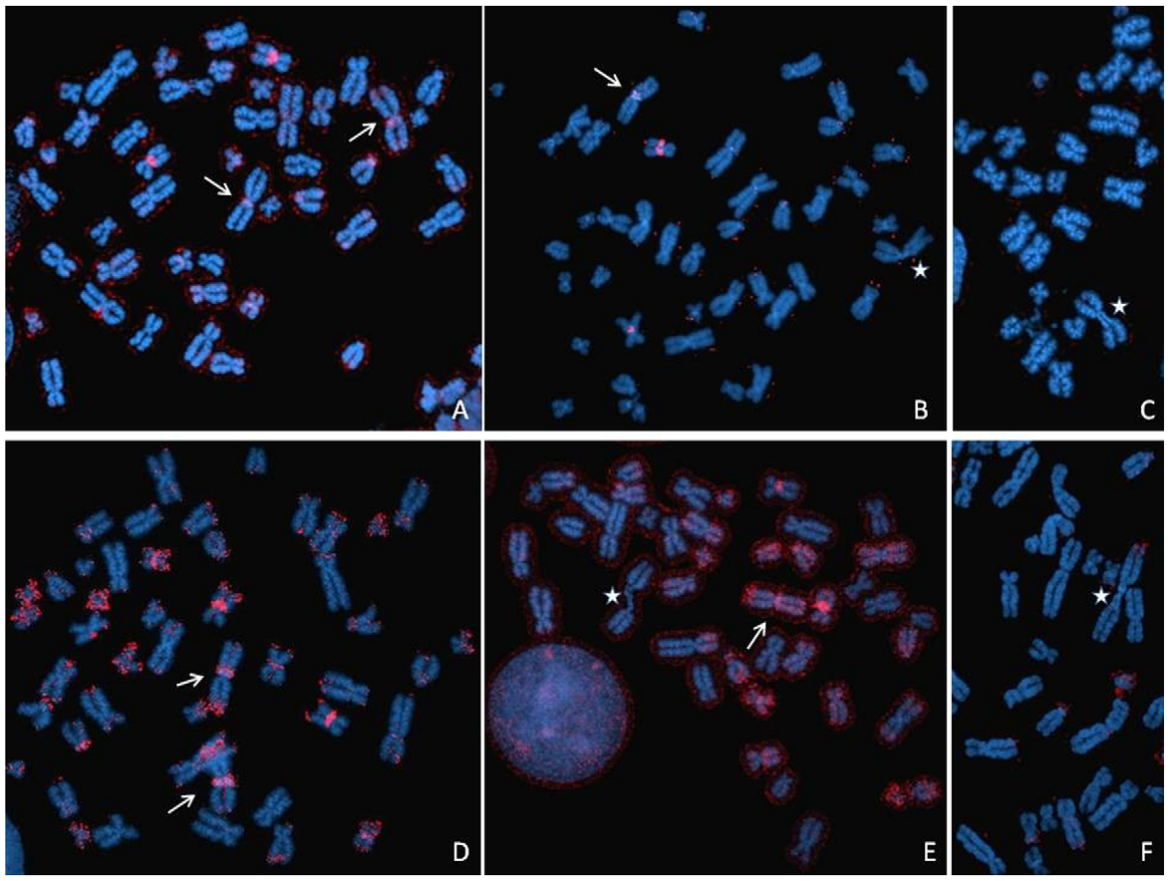
Changes in chromosomal methylation patterns upon treatment with 5-azacytidine. Control cells were treated with the demethylating agent 5-azacytidine and subsequently immunostained with a monoclonal antibody against 5-Methylcytidine (red signal). A significant variability in terms of extent and distribution of DNA methylation within the cell populations (Control 1: A, B and C; Control 2: D, E and F) was observed, with some metaphases and nuclei showing almost no methylation at all and others showing still substantial methylation, particularly on the compact heterochromatic areas (white arrows). However, stretched or decondensed heterochromatin in metaphase appeared to be consistently demethylated (white stars). Arrows and stars point specifically to chromosome 1 juxtacentromeric heterochromatin.

We first established the effect of the demethylating treatment on metaphase chromosomes by incubating unsynchronised B-lymphoblastoid cell lines from Control 1 and 2 with 5-azacytidine for 18 hours, following the protocol previously described by Ji et al. [25]. We compared metaphase spreads from the 5-azacytidine treated and non-treated cultures and, we observed that following the 5-azacytidine treatment, the otherwise normal control cells displayed decondensation of the chromosome 1 juxtacentromeric region in 10–15% of metaphases analysed (Figure S2 and Figure S3). We also carried out an immunostaining test with a monoclonal antibody against 5-Methylcytidine to detect changes in the chromosomal methylation patterns, with particular focus on the chromosome 1 heterochromatin. We observed that, following the 18 hour treatment with 5-azacytidine, there was a significant intercellular variability in terms of extent and distribution of DNA methylation, with some metaphases and nuclei showing almost no methylation at all and others showing still substantial methylation, particularly on the compact heterochromatic areas. However, most importantly, the stretched or decondensed juxtacentromeric heterochromatin appeared to be consistently demethylated (Fig. 9).

We then proceeded with observations on the chromosome 1 juxtacentromeric heterochromatin configuration in interphase and comparative assessments of the heterochromatin areas in ICF Patient 1 and 2, Control 1 and 2 and DO208915 B-lymphoblastoid cells before and after treatment with 5-azacytidine. Measurements were performed as described previously (Table 1). In the Kolmogorov-Smirnov test, the control cells showed a significant reduction in the nuclear area occupied by the heterochromatin after treatment with 5-azacytidine (D = 0.1697, P<0.001). However, the area of chromosome 1 juxtacentromeric heterochromatin is significantly smaller in non-treated ICF cell lines than 5-azacytidine treated control cell lines (D = 0.2601, P<0.001). There was also a significant difference in the areas of chromosome 1 juxtacentromeric heterochromatin between treated and non-treated ICF cell lines (D = 01341, P = 0.001).

**Table 1:** Heterochromatin configuration before and after 5-azacytidine treatment.

Cell Line	Mean Area (µm^2^)	SD (µm^2^)	N
ICF Patient 1 (5-azacytidine)	1.17	0.47	166
ICF Patient 1 (non-treated)	1.38	0.82	322
Control 1 (5-azacytidine)	1.89	1.08	178
Control 1 (non-treated)	2.86	1.69	297
ICF Patient 2 (5-azacytidine)	1.64	0.67	163
ICF Patient 2 (non-treated)	1.78	0.75	382
Control 2 (5-azacytidine)	2.37	1.13	197
Control 2 (non-treated)	2.46	1.49	309
DO208915 (5-azacytidine)	2.02	0.87	182
DO208915 (non-treated)	2.35	0.5	262

Area measurements of chromosome 1 juxtacentromeric heterochromatin, as defined by hybridisation with the classical satellite DNA probe D1Z1, were carried out on cells from ICF Patients 1 and 2, Controls 1 and 2, and DO208915, a third and unrelated control cell line, before and after treatment with the demethylating agent 5-azacytidine (SD = standard deviation; N = total number of measurements).

To investigate whether 5-azacytidine could also affect the positioning of the heterochromatin within the nuclear space, the average distance of chromosome 1 juxtacentromeric regions from the extreme nuclear periphery was measured and compared before and after treatment with 5-azacytidine. Measurements were carried out as described previously (Table 2). There was no significant difference between treated and non-treated ICF cell lines (D = 0.0708, P = 0.186) and no significant difference between the treated and non-treated control cell lines (D = 0.0727, P = 0.98). However, the statistically significant difference in the distance of chromosome 1 juxtacentromeric heterochromatin from the extreme nuclear periphery previously observed when comparing ICF and control cells was maintained even after treatment of the control cells with 5-azacytidine (D = 0.1020, P = 0.009).

**Table 2:** Heterochromatin positioning before and after 5-azacytidine treatment.

Cell Line	Distance	Radius	Ratio	SD	N
ICF Patient 1 (5-azacytidine)	4.12	10.01	0.412	2.16	208
ICF Patient 1 (non-treated)	5.24	11.39	0.460	2.90	311
Control 1 (5-azacytidine)	3.62	10.82	0.335	2.43	220
Control 1 (non-treated)	4.70	11.71	0.402	2.91	328
ICF Patient 2 (5-azacytidine)	5.53	12.55	0.441	2.70	196
ICF Patient 2 (non-treated)	4.75	9.29	0.512	2.37	358
Control 2 (5-azacytidine)	4.52	13.13	0.345	2.57	178
Control 2 (non-treated)	3.22	8.87	0.364	2.27	360
DO208915 (5-azacytidine)	4.59	14.05	0.327	2.43	201
DO208915 (non-treated)	3.77	11.18	0.337	2.62	259

The average distance between the centroid of the chromosome 1 juxtacentromeric heterochromatin signal, as defined by hybridisation with the classical satellite DNA probe D1Z1, to the extreme nuclear periphery was measured in cells from ICF Patients 1 and 2, Controls 1 and 2, and DO208915, a third and unrelated cell line, before and after treatment with the demethylating agent 5-azacytidine (Distance = Mean Distance in µm; Nuclear Radius = Mean Nuclear Radius in µm; Ratio = Mean Distance/Nuclear Radius; SD = standard deviation; N = total number of measurements).

Comparative analysis of the nuclear architecture parameters used so far in our investigation, carried out in different cell lines before and after treatment with the global demethylating agent 5-azacytidine, shows a less conspicuous, but still significant remodelling of chromosome 1 juxtacentromeric heterochromatin, resulting in an altered configuration of this genomic region in both metaphase and interphase similar to what observed in ICF cells. In contrast, positioning of the heterochromatin in relation to the nuclear periphery seems unaffected by the demethylating treatment.

### Analysis by quantitative PCR identifies significant differences in the amount of classical satellite DNA between the different cell lines

To compare the abundance of the two classical satellite DNA families comprising the bulk of the chromosome 1 juxtacentromeric heterochromatin, Real Time PCR experiments with chromosome 1-specific satellite 2 and 3 primers were carried out in ICF cells and controls (Table 3). ICF Patient 1 has about thirty times less satellite 2, and two times less satellite 3 than Control 1. However, when compared to the unrelated control D0208915, ICF patient 1 seems to have two times less satellite 2, but no significantly different amount of satellite 3. ICF Patient 2 has two times less satellite 2 than Control 2, but eleven times more than the unrelated control D0208915. For the satellite 3, there is no significant difference between ICF Patient 2 and either control.

**Table 3:** Relative quantitation of satellite 2 and 3 using the comparative C^t^ method.

Comparisons	*SAT2*	*SAT 3*
Control 1 vs ICF Patient 1	28.9	2.02
D0208915 vs ICF Patient 1	2.4	Not significant
Control 2 vs ICF Patient 2	2.28	Not significant
D0208915 vs ICF Patient 2	−11	Not significant

The amount of satellite 2 and 3 present in each patient is tabulated as –fold increase with respect to different controls. Only values showing statistical significance are shown.

While the abundance of the classical satellite 3 appears more or less constant, the marked inter-individual differences observed for the classical satellite 2 are consistent with the length-polymorphism of this repetitive DNA family and heteromorphism of the juxtacentromeric heterochromatin. We show that both ICF patients present less satellite 2 repeats than their relative controls. However, either combining the two satellites results or considering them individually, our results show no statistically significant correlation between satellite DNA abundance and heterochromatin areas measurements in interphase (Pearson Product-Moment Correlation combined: R = 0.411 P = 0.49; Satellite 2 R = 0.43, P = 0.47; Satellite 3 R = −0.206, P = 0.74 respectively).

### Analysis by Real Time RT-PCR confirms altered expression of *BTG2*, *CNN3*, *ID3*, *RGS1* and *F13A1* in ICF

Real-time RT-PCR was performed to compare the relative expression levels of four genes from chromosome 1 (*BTG2*, *CNN3*, *ID3*, *RGS1*) and one gene from chromosome 6 (*F13A1*) in the cell lines under investigation. Relative gene expression of the above genes was compared between ICF Patient 1 and Control 1, and ICF Patient 2 and Control 2. β-actin was used as a normalisation gene. The results are summarised in Table 4. Our Real-time RT-PCR results show altered gene expression in ICF cells, more specifically up-regulation of *CNN3*, *RGS1* and *F13A1* and down-regulation of *BTG2* and *ID3*.

**Table 4:** Gene expression analysis by real-time RT-PCR.

Gene	Primers	Primer sequences	Cell lines	Fold-difference
***BTG2*** ** (1q32.1)**	BTG2-RT 3f/3r	(5′-gaaccgacatgctccc-3′) (5′-cagtggtgtttgtagtga-3′)	ICF 1 vs. C 1	−2.142 (p = 0.001)
			ICF 2 vs. C2	−5.460 (p = 0.013)
	BTG2-RT 4f/4r	(5′-aataaaagccaaacct-3′) (5′-gctttccacttttctcca-3′)	ICF 1 vs. C 1	−2.071 (p = 0.004)
			ICF 2 vs. C 2	−2.624 (p = 0.001)
***CNN3*** ** (1p21.3)**	CNN3-RT 3f/3r	(5′-taacattacagccggtgg-3′) (5′-aggagcagcacagtatt-3′)	ICF 1 vs. C 1	+2.046 (p = 0.001)
			ICF 2 vs. C2	+4.854 (p = 0.001)
	CNN3-RT 4f/4r	(5′-gcaattggatagaagagg-3′) (5′-ggactcgttgaccttct-3′)	ICF 1 vs. C 1	+2.104 (p = 0.001)
			ICF 2 vs. C2	+2.019 (p = 0.001)
***ID3*** ** (1p36.12)**	ID3-RT 4f/4r	(5′-caaactatgccaaggcg-3′) (5′-cgcattgttacagaaagtca-3′)	ICF 1 vs. C1	−2.432 (p = 0.012)
			ICF 2 vs. C 2	−2.556 (p = 0.001)
***RGS1*** ** (1q31.2)**	RGS1-RT 3f/3r	(5′-acagatagtatcaagcgca-3′) (5′-gcgcctggataactttc-3′)	ICF 1 vs. C1	+2.847 (p = 0.001)
			ICF 2 vs. C2	+4.512 (p = 0.004)
	RGS1-RT 4f/4r	(5′-aagcgcagaaggaatg-3′) (5′-gcgcctggataactttca-3′)	ICF 1 vs. C1	+2.898 (p = 0.001)
			ICF 2 vs. C2	+2.107 (p = 0.005)
***F13A1*** ** (6p25.1)**	F13A1-RT 1f/1r	(5′-cgtcaacctgcaagag-3′) (5′-cgaccaatgacgtattcc-3′)	ICF 1 vs. C1	+1.472 (p = 0.001)
			ICF 2 vs. C 2	+3.517 (p = 0.001)
	F13A1-RT 1f/2r	(5′-cgtcaacctgcaagag-3′) (5′-acatagaaagactgccct-3′)	ICF 1 vs. C 1	+5.378 (p = 0.009)
			ICF 2 vs. C2	+2.843 (p = 0.001)

A relative expression study for genes *BTG1*, *CNN3*, *ID3*, *RGS1* and *F13A1* in ICF patients versus controls was performed using Real-Time Reverse-Transcription PCR (RT-PCR). Relative expression levels of genes between ICF patients and controls were calculated using the equation described in Materials and Methods.

### Analysis by MALDI-TOF mass spectrometry reveals no significant methylation differences in CpG islands of gene promoters between ICF and controls

DNA methylation analysis of CpG islands in the promoter region of three of the genes under investigation, more precisely *BTG2*, *CNN3* and *ID3*, was performed to examine whether altered expression in ICF cells may have been caused by changes in promoter methylation. No CpG islands are present in the promoters of *RGS1* and *F13A*.

The analysis was performed on bisulfite treated DNA from ICF Patient 1 and 2, Control 1 and 2, and DO208915 cell lines using a sequencing by fragmentation assay for quantitative methylation analysis [27]; this assay is based on RNA transcription and base -specific cleavage. Multiple CpG sites can be detected in a single experiment and altered methylation is detected as a G/A change on the reverse strand. The results for all three genes show that there are no significant differences in overall promoter CpG island methylation between ICF cells and controls. At all CpG sites analysed, very low and comparable levels of methylation were found for all ICF patient and control cell lines. The data are summarised in the EpiGrams for each gene (Figure S4).

These results show that up-regulation of *CNN3* and down-regulation of *BTG2* and *ID3* are not linked to obvious changes in the DNA methylation of their promoters.

### Intra-nuclear positioning of abnormally expressed genes and co-localisation with juxtacentromeric heterochromatin

The association of the above genes with the extreme nuclear periphery and relative positioning to the chromosome 1 juxtacentromeric heterochromatin were assessed by co-hybridising each of the four BAC clones, containing the chromosome 1 genes *BTG2*, *CNN3*, *ID3* and *RGS1*, with the classical satellite DNA probe D1Z1 to interphase nuclei obtained from ICF patient and control B-lymphoblastoid cells. A BAC clone containing the chromosome 6 gene *F13A1* was also co-hybridised with D1Z1 as well as with the chromosome 6 alpha satellite DNA probe D6Z1 and the chromosome 16 classical satellite 2 DNA probe D16Z3. These were control experiments designed to respectively identify: (a) possible involvement of chromosome 1 juxtacentromeric heterochromatin in inter-chromosomal associations, (b) similarity of behaviour in terms of inter-chromosomal gene-heterochromatin associations between chromosome 1 and 16, and, (c) intra-chromosomal associations involving a chromosome not specifically affected by molecular and cytological changes in ICF syndrome.

**Figure 10:**
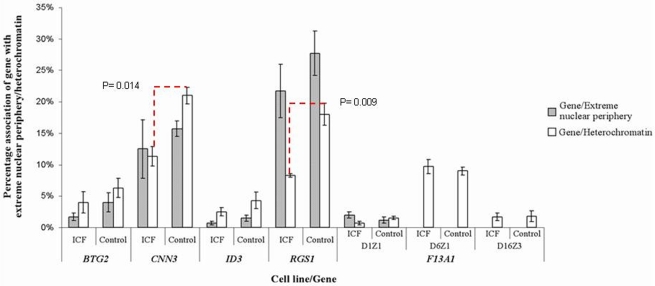
*BTG2*, *CNN3*, *ID3 RGS1* and *F13A1*: Association with the extreme nuclear periphery and the juxtacentromeric heterochromatin. Two-colour FISH using separate probes to identify the gene and the juxtacentromeric or centromeric heterochromatin was performed on quiescent cells from ICF patient 1 and Control 1, and cycling cells from ICF Patient 1 and 2 and Control 1 and 2. Two parameters were investigated; the association of the gene with the extreme nuclear periphery and the association of the gene with heterochromatin. From left to right, the graph shows the association of *BTG2*, *CNN3*, *ID3*, *RGS1* and *F13A1* with the extreme nuclear periphery in ICF cells and controls (shaded bars), and association with chromosome 1 juxtacentromeric heterochromatin (D1Z1) in ICF and controls (white bars). The association of *F13A1* with chromosome 6 centromeric heterochromatin (D6Z1) and chromosome 16 juxtacentromeric heterochromatin (D16Z3) in ICF cells and controls is also shown in the right-most four columns of the graph. A minimum of 250 observations were carried out for each experiment. The extent of co-localisation for *CNN3* and *RGS1* with the chromosome 1 juxtacentromeric heterochromatin was found to be significantly different when comparing ICF and control cells (χ^2^ = 6.028, P = 0.014 for *CNN3* and χ^2^ = 6.775, P = 0.009 for *RGS1*).

Association of each gene with the extreme nuclear periphery was compared in ICF Patient 1 and Control 1, and ICF Patient 2 and Control 2. A signal was considered to be positioned at the extreme nuclear periphery if any part of it appeared to associate with the nuclear rim, as defined by the edge of the DAPI staining. A minimum of 250 observations were carried out per probe per cell line (Figure 10).

For three of the genes under investigation, *BTG2* and *ID3* from chromosome 1 and *F13A1* from chromosome 6, the degree of association of each locus with the extreme nuclear periphery was practically negligible in both ICF cells and controls. The other two genes from chromosome 1 - *CNN3* and *RGS1* - showed a higher percentage of peripheral location, although, as before, no preferential positioning at the extreme nuclear periphery were evident for either loci. Statistical analysis confirmed that the intra-nuclear positioning of all genes analysed, as defined by association with the extreme nuclear periphery, is not altered in ICF cells as the differences between ICF and controls are not significant when using a Chi-squared test (χ^2^ = 1.042, P = 0.307 for *BTG2*; χ^2^ = 0.314, P = 0.575 for *CNN3*; χ^2^ = 1.010, P = 0.315 for *ID3*; χ^2^ = 1.786, P = 0.181 for *RGS1*; χ^2^ = 1.010, P = 0.315for *F13A1*).

**Figure 11:**
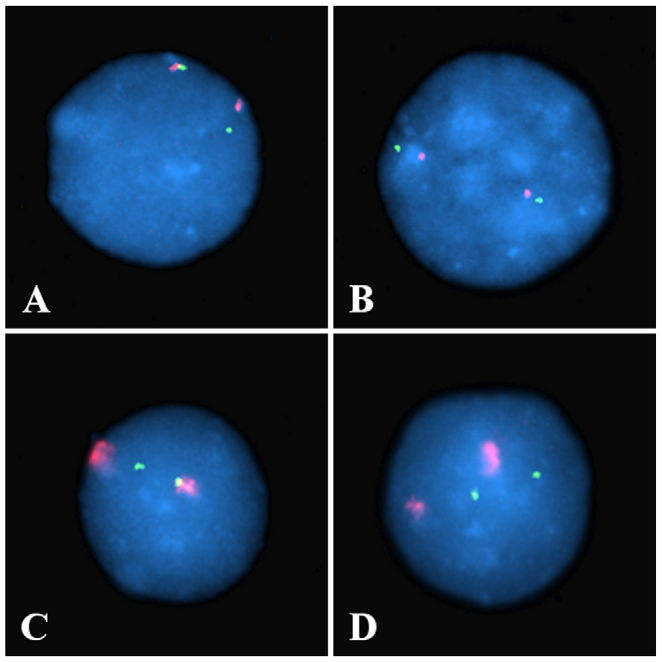
Gene-heterochromatin co-localization assessment. Co-localisation was assessed by identifying gene signals (green) showing any degree of overlap with the classical satellite DNA signal (red). The co-localisation assessment was carried out independently from the heterochromatin spatial configuration (“conventional” in A and B, and “compact” in C and D) and the intra-nuclear positioning of both gene and heterochromatic signals.

The positioning of the four genes from chromosome 1, *BTG2*, *CNN3*, *ID3* and *RGS1*, in relation to chromosome 1 heterochromatin was also analysed. Co-localisation was assessed by identifying on cells of ICF Patient 1 and Control 1, and ICF Patient 2 and Control 2, gene signals that showed any degree of overlap with the satellite DNA signal. The co-localisation assessment was carried out independently from the heterochromatin spatial configuration and the intra-nuclear positioning of both gene and heterochromatic signals (Fig. 11). A minimum of 250 observations were carried out per probe per cell line (Figure 10).

*BTG2* and *ID3*, the two genes from chromosome 1showing low association with the extreme nuclear periphery, are also characterised by a comparably low extent of co-localisation with the heterochromatin. On the contrary, *CNN3* and *RGS1*, the two genes from chromosome 1 showing a relatively higher extent of association with the extreme nuclear periphery, are also characterised by a relatively higher extent of co-localisation with the heterochromatin.

For the two down-regulated genes, *BTG2* and *ID3*, the extent of co-localisation with chromosome 1 heterochromatin was not significantly different between ICF cell and controls, using a Chi-squared test (χ^2^ = 0.709, P = 0.400 for *BTG2* and χ^2^ = 0.260, P = 0.610 for *ID3*). However, the extent of co-localisation for *CNN3* and *RGS1*, the other two genes from chromosome 1 that appear to be up-regulated, was significantly different in ICF and control cells (χ^2^ = 6.028, P = 0.014 for *CNN3* and χ^2^ = 6.775, P = 0.009 for *RGS1*).

The extent of co-localisation between *F13A1*, the over-expressed gene from chromosome 6, and both chromosome 1 and chromosome 16 juxtacentromeric heterochromatic regions was negligible and there were no significant differences between ICF cells and controls (χ^2^ = 0.510, P = 0.475; χ^2^ = 0.000, P = 1.000). The extent of co-localisation between *F13A1* and the chromosome 6 centromeric region, as defined by hybridisation with the alpha-satellite DNA probe D6Z1, was also assessed and showed no statistical significance (χ^2^ = 0.122, P = 0.727).

In conclusion, although the genes under investigation present a variable extent of association with the extreme nuclear periphery, none of them shows preferential positioning there, and, in this respect, there are no significant differences between ICF and control B-cells. However, in terms of co-localisation with the juxtacentromeric heterochromatin, the inter-genic variability is more pronounced, with two of the genes from chromosome 1 – *CNN3* and *RGS1* - showing a greater extent of co-localisation with the chromosome 1 juxtacentromeric heterochromatin. Most importantly, for these two loci the extent of gene-heterochromatin co-localisation is significantly reduced in the ICF cells in which these genes appear over-regulated when compared to the control cells.

## Discussion

The complexity of ICF, in particular the combination of phenotypic variability and genetic heterogeneity that characterises it, has intrigued geneticists and cell biologists since this syndrome was initially identified. Over the years, numerous and diverse investigations have yielded interesting insights into its pathogenesis and prompted a substantial amount of speculation on the relationship between methylation defects, chromatin abnormalities and clinical symptoms that characterise this complex disorder [2], [14], [18], [28].

Investigations on the chromosomal disturbances in ICF have been so far conducted predominantly on metaphase chromosomes and, although there has been a number of observations carried out in interphase [5], [29], [30], [31], [32], [33], [34], [35], our study provides the first extensive and statistically substantiated analysis on the nuclear architecture of genes and heterochromatic regions in this syndrome. In particular, we have examined the large-scale organisation of chromosome 1 and chromosome 16 juxtacentromeric heterochromatic regions, their intra-nuclear positioning, and their co-localisation with five specific genes, four from chromosome 1 and one from chromosome 6, on which we have concurrently conducted expression and methylation analysis. These genes express proteins with different functions, ranging from cell growth and differentiation to blood coagulation and to association with the cytoskeleton and we selected them on the basis of their chromosomal location, within a collection of genes previously reported to be abnormally expressed in ICF [21]. The investigations have been carried out in parallel in two unrelated patients, one with Type 1 ICF and the second with Type 2 ICF, both presenting the hypomethylation of the classical satellite 2 DNA typical of this syndrome [8]. One unrelated and two related controls (unaffected parents of the ICF patients) have been also included in the study.

The comparative analysis of the large-scale organisation of the juxtacentromeric heterochromatin, undertaken by FISH analysis on interphase nuclei, has disclosed intriguing differences between ICF and control cells. To begin with, observations at the microscope on a cell-by-cell basis and 2D measurements of the areas occupied by chromosome 1 heterochromatin have revealed that these areas were on average significantly smaller in nuclei from the patient cell lines when compared to the controls, suggesting an altered intra-nuclear arrangement of this specific genomic region in ICF. These unexpected conclusions were also confirmed by heterochromatin volume measurements obtained by 3D FISH, a cytological hybridisation procedure acknowledged to better preserve nuclear architecture, followed by confocal analysis. A similar reduction in the size of the heterochromatin hybridisation signals in ICF cells was also observed for chromosome 16, although when analysed statistically the difference was shown to be not significant.

Because of the observed inter-nuclear variability, we decided to investigate a possible connection between heterochromatin remodelling and progression through the cell-cycle. Based on our FACS analysis and our results on BrdU pulse-labelled cells, we were able to demonstrate that dissimilar percentages of different heterochromatin configurations within each cell population cannot be attributed to differences in cell-cycle progression.

We showed that a downsized configuration of the heterochromatin in interphase, similar to that observed in ICF, can be partly reproduced in control cells by treatment with 5-azacytidine. This demethylating agent had been previously used to reproduce *in vitro* some of the defects observed on metaphase chromosomes in ICF syndrome [24], [25], [26]. Our results provide for the first time evidence that hypomethylation of classical satellite DNA sequences, as well as contributing to promote the abnormal chromatin structure of the juxtacentromeric regions in metaphase previously described in these patients [8], also affects the large-scale organisation of the same heterochromatic regions in interphase.

Our unexpected findings of an apparently more compact configuration of the chromosome 1 juxtacentromeric heterochromatin in ICF B-cell nuclei do not concur with the consensus – based on the generally acknowledged parallel between DNA methylation and chromatin compaction [rev. in [36], [37]] and also supported by earlier observations on ICF cells [29], [34] - that decondensation and stretching of the heterochromatin, as observed in metaphase, should also be expected in interphase. However, we feel confident with the extent and variety of our investigations and the robust statistical analysis that supports our findings. Interestingly, the canonical view of a direct correspondence between methylation and chromatin condensation has also been challenged by Gilbert and co-authors. [38], who, by using mutant mouse embryonic stem cells completely lacking in DNA methylation, have recently shown that chromatin compaction, as assayed by nuclease digestion and sucrose gradient sedimentation, is not affected in these cells, their results underlining the complexity of the relationship between DNA methylation and chromatin structure.

Because of the intrinsic resolution limits of the microscopy techniques and the recurring concerns in the field of chromosome biology on the effects of cell fixation on the “live” properties of the chromatin fibres in the nucleus, in particular in 2D FISH procedures [39], we are aware that any attempt to explain the observed altered arrangement of the juxtacentromeric heterochromatin in ICF nuclei should be particularly cautious. However, we speculate that the downsizing of the heterochromatin signal could be partly the effect of a collapse in the folding of the chromatin fibre caused by changes in the steric properties of the hypomethylated satellite DNA and the resulting destabilization of the chromatin structure, probably rendered more obvious by the fixation procedures.

While investigating the altered heterochromatin organisation in ICF, a possible linkage with the heteromorphism of the heterochromatin deserves also consideration, as the downsizing of the heterochromatic signal in interphase in ICF cells could simply occur as the result of a substantial reduction in the number of their classical satellite repeats. An interesting study by Blasco and collaborators [40] has reported a reduction in centromeric repeats in mouse cells lacking the Dnmt3a and Dnmt3b DNA methyltransferases, suggesting DNA methylation at the centromeric heterochromatin to be an important mechanism to suppress “illicit” centromere mitotic recombination and to maintain centromere integrity. The hypothesis that a variation in the amount of juxtacentromeric heterochromatin repeat DNA or satellite DNA length polymorphism may underlie the phenotypic variability observed in ICF was formulated by Luciani and co-authors in the context of their investigations on HP1 sub-cellular distribution in ICF [30]. However, they speculated the presence of longer stretches of 1q or 16q repeats in the disorder.

The analysis of the classical satellite DNA that we carried out by quantitative PCR has indeed confirmed marked inter-individual dissimilarities and a significant difference in enrichment of classical satellite 2 repeats in ICF patients when compared to their controls, with both patients showing fewer classical satellite 2 DNA repeats than their respective controls. However, our analysis has also highlighted the absence of a direct correlation between satellite DNA length-polymorphism and heterochromatin configuration. Taken all together, our observations point towards an intermediate scenario in which both DNA hypomethylation and differences in the copy number of classical satellite sequences contribute to the altered spatial organisation of the juxtacentromeric heterochromatin in ICF.

Having established the existence of consistent and quantifiable differences in the large-scale organisation of the juxtacentromeric heterochromatin in ICF, we proceeded to investigate possible changes in the intranuclear positioning of this genomic region in this syndrome, using as a spatial reference the extreme periphery. Although its role in actively regulating gene expression remains unproven, the nuclear periphery is generally considered a transcriptionally silent “address” within the nuclear volume, characterised in yeast by the high concentration of silencing *sir* proteins [41] and in higher eukaryotes by poor gene density [42], [43], [44] and high concentration of non-transcribed sequences [45]. Also, repositioning of silent genes from the nuclear interior to the nuclear periphery has been observed in few instances [46], [47], [48], [49].

Our measurements of the distance between chromosome 1 heterochromatin and the nuclear rim have revealed that the extent of association with the nuclear periphery is reduced in ICF B-cells, suggesting a specific re-positioning of this genomic region to a more internal location within the nuclear space. Based on the differential distribution of early and late-replicating chromatin within the nucleus [50], [51], our findings on the relocation of the heterochromatin away from the extreme nuclear periphery to a more internal position agree with the advanced replication of the hypomethylated satellite 2 previously reported in ICF [8]. Evidence for a similar repositioning of the chromosome 1 juxtacentromeric heterochromatin within the nuclear volume, following treatment with the histone deacetylase inhibitor trichostatin A (TSA), was published before [52].

Our findings on the altered large-scale organisation and intra-nuclear positioning of chromosome 1 juxtacentromeric heterochromatin in ICF are particularly significant in the light of the mounting experimental evidence suggesting chromosome band 1q12 to be the core of a nuclear domain with functional significance, with earlier investigations showing physical association of this genomic region with the human polycomb group complex [53], and also with the oncogenic transcriptional regulator TLX1/HOX11 in leukemic T-cells [54]. In ICF cells the 1qh satellite DNA is associated in G2 with a giant HP1-PML nuclear body [31].

In order to explore the existence of a possible link between altered heterochromatin organisation and changes in gene expression in ICF, we investigated the intra-nuclear positioning of four specific genes from chromosome 1 (*BTG2*, *CNN3*, *ID3* and *RGS1*), using as a spatial reference their association with the extreme nuclear periphery, as well as their co-localisation with chromosome 1 heterochromatin. The genes were selected from a collection of genes previously reported to be abnormally expressed in ICF [21]. We were also interested in identifying possible long-range interchromosomal gene-heterochromatin associations, therefore we included in the analysis *F13A1*, a gene mapping on 6p25-24, also previously reported to be abnormally expressed in ICF [21]. In parallel to the cytological investigations, relative gene expression analysis was carried out by Real Time RT-PCR. Our experiments showed comparative up-regulation of *CNN3*, *RGS1* and *F13A1* and down-regulation of *BTG2* and *ID3* in our ICF cell lines, confirming previous results obtained by microarray analysis [21].

We also tested for a direct role of methylation on gene expression changes by carrying out a comparison of CpG islands in the promoter regions of three of the genes under examination, namely *CNN3*, *BTG2* and *ID3*. Our methylation analysis, carried out by base-specific cleavage and mass spectrometry, established that the genes were largely unmethylated and detected no significant changes in ICF cells. The promoter regions of *RGS1* and *F13A1*, the other two genes under investigation not included in our methylation analysis due to the absence of CpG islands in their promoters, had previously shown no ICF-linked changes in the overall promoter methylation [21].

In terms of nuclear positioning, our observations show two of the genes analysed – *RGS1* and *CNN3* – to be not exclusively, but more frequently associated with the extreme nuclear periphery than the other three genes under investigation, for which the degree of association with the nuclear rim was negligible. However, no significant differences were observed when the positioning of each of the genes in relation to the extreme nuclear periphery was compared between ICF cells and controls.

Results that are more relevant were provided by our analysis of genes and chromosome 1 juxtacentromeric heterochromatin co-localisation, as *RGS1* and *CNN3*, the two up-regulated genes from chromosome 1, showed a significant reduction in their extent of co-localisation with juxtacentromeric heterochromatin in ICF nuclei when compared to controls. Correlation between gene silencing and localisation to transcriptionally repressive heterochromatic compartments has been reported in mouse cycling lymphocytes [55], [56], [57], human and mouse erythroid cells [58], [59], [60] and retinoblastoma cells [61]. More recently, a link between centromeric recruitment and establishment of allelic exclusion at the immunoglobulin heavy-chain gene in mouse B-cells was also reported [62]. Therefore, it is conceivable that *RGS1* and *CNN3* are normally silenced in B-cells through association with the heterochromatin and this association is disrupted in ICF.

In contrast to what was observed for *RGS1* and *CNN3*, the extent of co-localisation between chromosome 1 juxtacentromeric heterochromatin and *BTG2* and *ID3*, the two genes showing down-regulation in ICF but no significant changes in promoter CpG islands methylation, was negligible and there were no significant differences between ICF cells and controls. These findings solicit further investigations into different aspects of nuclear architecture and other possible epigenetic mechanisms likely to affect the regulation of these two genes.

In conclusion, we suggest that in ICF the length and hypomethylation of the classical satellite 2 DNA, the main component of the juxtacentromeric heterochromatin of chromosomes 1,and 16, are not only responsible for the centromeric abnormalities generally observed in metaphase, but also affect the three-dimensional organisation of the heterochromatin in interphase. This is based on our findings – partly reproducible in control cells by demethylating treatment - that in ICF B-cell nuclei the chromosome 1 juxtacentromeric heterochromatin appears significantly smaller in volume and more internally positioned within the nuclear space. On the basis of our observations on the changes in the extent of co-localisation of two up-regulated genes (*CNN3* and *RGS1*) and chromosome 1 juxtacentromeric heterochromatin, we also postulate that, by affecting long-range gene-heterochromatin associations, the altered intra-nuclear arrangement of the hypomethylated classical satellite sequences interferes with heterochromatin mediated gene silencing and contributes to some of the changes in gene expression observed in ICF.

Our findings support earlier suggestions of an epigenetic impact of chromatin and chromosomal changes in ICF syndrome and present an example of how human diseases can provide ideal model systems to investigate the functional significance of nuclear architecture.

## Materials and Methods

### Cell lines

The ICF B-lymphoblastoid cell line GM08714A, and a control cell line generated from the patient's mother, GM08728, were obtained from the Coriell Cell Repositories (USA) http://ccr.coriell.org/. A second ICF B-lymphoblastoid cell line, LB188, and a control cell line from the patient's mother, LB290, had been previously established and described [63]. An additional control cell lines used for the study was the B-lymphoblastoid cell line, DO208915 (European Collection of Cell Cultures, UK). For simplicity purposes, in the paper the ICF cell line GM08714A is referred to as ICF patient 1 and the ICF cell line LB188 as ICF patient 2. The control cell line GM08728 is referred to as Control 1, and LB290 as Control 2. ICF patient 1 is a compound heterozygote for mutations in *DNMT3B*, carrying a G>A transition at nucleotide 1807 on one allele, and a G>A transition within intron 22, 11 nucleotides 5′ of a splice acceptor site on the other allele, and has Type 1 ICF syndrome. ICF patient 2 does not carry a mutation in *DNMT3B* and has Type 2 ICF syndrome. Data on the hypomethylation of satellite 2 in both patient cell lines can be found in Hassan et al. 2001 [8] (wherein GM08714 is referred to as PT4 and LB188 is referred to as PT12).

### Cell culture and slides preparation

Cells were cultured in suspension in RPMI-1640 medium (Sigma-Aldrich, UK) supplemented with 10% foetal bovine serum (Sigma-Aldrich) and 1% L-Glutamine at 37°C in a 5% CO_2_ incubator. Slow-growing cultures, enriched in G0/G1 cells, were obtained by incubating the cells with no serum for 72 hours. For cell-cycle investigations, cells were pulse-labelled with 10 µM 5-Bromo-2-deoxyuridine (BrdU) (Sigma-Aldrich) for 30 minutes prior to cell harvesting. For the demethylating agent treatment, 5-azacytidine (Sigma-Aldrich) was added to the cell cultures at a final concentration of 0.5 µM followed by incubation at 37°C for 18 hours, a wash and incubation in normal conditions for 72 hours prior to cell harvesting, as previously described [25]. To obtain metaphase chromosomes, thymidine (Sigma-Aldrich) was added to each culture at a final concentration of 0.3 mg mL^−1^ and incubated at 37°C for 17 hours. 10 minutes prior to harvest, Colcemid (Invitrogen, UK) was added at a final concentration of 0.2 µg mL^−1^. The cells were centrifuged, resuspended in prewarmed hypotonic solution (0.075 M potassium chloride) for 5 minutes and fixed in three changes of 3∶1 methanol: acetic acid. Slides were prepared according to standard procedures. Metaphase chromosomes obtained from cultures treated with 5-azacytidine were harvested without thymidine. For interphase preparations no thymidine or Colcemid were used. For 3D FISH analysis, cells were resuspended in 1× PBS at a density of 2×10^6^ cells mL^−1^. 200 µL of the cell suspension was pipetted onto a poly-lysine coated slide (VWR International, UK) and the slide incubated in a moist chamber for 1 hour at 37°C. Following incubation, the slides were processed by washing in 1× PBS on ice for 5 minutes and then in CSK/TX (0.1 M NaCl; 0.3 M Sucrose; 0.003 M MgCl2; 0.01 M Pipes; 0.5% Triton X-100). The cells were fixed in 4% formaldehyde/1× PBS for 5 minutes at room temperature, washed in 1× PBS and permeabilised in 0.5% Triton X-100/1× PBS for 20 minutes at room temperature. The slides were again washed in 1× PBS and then incubated in 0.1 M HCl for 10 minutes at room temperature. After a final wash in 1× PBS, the slides were stored in 70% ethanol.

### Probes

BAC clones containing genes *BTG2* (RP11-134p9), *CNN3* (RP4-639p13), *ID3* (RP1-150o5) and *RGS1* (RP5-1011o1) were obtained from the Sanger Institute (Cambridge, UK). A DNA preparation of the BAC containing gene *F13A1* (287k15) was obtained directly from the Genomics Core Group, Wellcome Trust Centre for Human Genetics, Oxford. BAC DNA extraction was carried out according to standard procedures. Prior to FISH analysis, the BAC clones were verified for gene content by Polymerase Chain Reaction (PCR) amplification. The primers (Table S1) were generated using “Primer 3” design software (http://frodo.wi.mit.edu/cgi-bin/primer3/primer3_www.cgi) based on the genomic sequences of *BTG2*, *CNN3*, *ID3* and *RGS1* obtained from the Human Genome Browser Gateway (http://genome.ucsc.edu/cgi-bin/hgGateway?org=human). Primers were synthesised by MWG Biotech (Germany). Directly labeled chromosome 1 classical satellite probe (D1Z1) (Qbiogene, UK) and chromosome 16 satellite 2 DNA probe (D16Z3) (Abbott Laboratories, UK) were used to visualise the juxtacentromeric heterochromatin. The chromosome 6 alpha satellite probe (D6Z1) (Qbiogene) was also used.

### Fluorescence *in situ* hybridisation (FISH)

80 ng of probe DNA - labeled with biotin-11-dUTP (Roche) using a nick-translation kit (Abbott Laboratories) - and 2 µg of human *C_0_t-1* competitor DNA (Invitrogen) were dried on a heating block at 65°C and resuspended in 1× hybridisation buffer (50% formamide, 1× SSC and 10% dextran sulphate) to a final concentration of 16 ng µL^−1^. Prior to hybridisation, the probes were denatured at 72°C for 10 minutes and pre-annealed at 37°C for 30 minutes. The chromosome 1 classical satellite probe (D1Z1), chromosome 16 satellite 2 DNA probe (D16Z3) and chromosome 6 alpha-satellite (D6Z1) probe were denatured at 85°C for 5 minutes and cooled on ice for 5 minutes prior to hybridisation. The slides were denatured in 70% formamide/0.6× SSC at 70°C for 2 minutes. Following hybridisation, in a moist chamber at 37°C overnight, the slides were washed in 50% formamide/1× SSC at 42°C for 10 minutes and 2× SSC at 42°C for 5 minutes. The biotinylated probes were detected with a layer of streptavidin conjugated FITC (Vector Laboratories, UK) when co-hybridised with red-labelled heterochromatin probes, or streptavidin-Texas Red (Invitrogen) when used with green heterochromatin probes. Slides were mounted with Vectashield (Vector Laboratories) containing 4′, 6-diamidino-2-phenylindole (DAPI) for nuclear staining. Prior to FISH, the BrdU pulse-labelled cells were treated with 0.5% Triton X-100 in 1× PBS for 10 minutes and then transferred to 0.1 M hydrochloric acid for 10 minutes at room temperature. The slides were then washed in 2× SSC for 5 minutes and then equilibrated in 50% formamide/2× SSC for at least 15 minutes prior to denaturation. BrdU labelling was detected using mouse anti-BrdU antibody (Roche) in 4% BSA in 1× PBS/0.1% Tween-20 and incubated at 37°C for 30 minutes. This was detected using goat anti-mouse Alexa 488 (Invitrogen). Slides were mounted with Vectashield containing DAPI.

### Immunofluorescence

Metaphase slides were denatured in 65% formamide, 2×SSC at 65°C for 30′, then dehydrated in ethanol series, and air-dried. The slides were then briefly washed in 2×SSC and incubated with blocking solution (1% non-fat dried milk in PBS/0.1% Tween-20) at 37°C. After 30 minutes a monoclonal antibody against 5-Methylcytidine (Eurogentec) (diluted 1∶100 in PBS/0.1% Tween-20) was applied to the samples and the slides were then incubated for 2 hours. The slides were finally washed in PBS three times for 10 minutes and incubated with a secondary antibody, Texas Red goat-anti mouse (Sigma) for 30 minutes. Slides were mounted with Vectashield containing DAPI.

### Image acquisition and analysis

FISH experiments were examined with a 100×, 1.3 NA oil-immersion objective lens fitted to an Olympus BX-51 epifluorescence microscope coupled to a Sensys charge-coupled device (CCD) camera (Photometrics, USA). Blue, green and red fluorescence images were taken as separate grey-scale images using the 83000 filter set manufactured by Chroma (USA) and then pseudo-coloured and merged using the software package Genus (Applied Imaging International, UK). Grey-scale images of the heterochromatin taken with Genus were imported into Volocity (Improvision, UK), and an image series created for each experiment. Using the Classifier feature, the areas of juxtacentromeric heterochromatin were measured using the percentage mode empirically set to a lower limit of 27% intensity, an upper limit of 100%, and to exclude areas smaller than 25 pixels and larger than 500 pixels (examples in Figure S5). Nuclei were scored randomly. Measurements form two independent experiments were obtained for each cell line and the mean areas calculated. Measurements of the volumes of D1Z1 were also obtained using Volocity. Z stacks generated by laser scanning confocal microscopy (Zeiss LSM510META) were imported into Volocity, where the Classifier feature, empirically set to threshold images at a lower limit of 14% intensity and upper limit of 100%, measured the volumes occupied by D1Z1 signal. Measurements of the distances of chromosome 1 and 16 juxtacentromeric heterochromatin from the extreme nuclear periphery were imported into the software package Volocity and an image series generated for each experiment. Using the VoxelSpy feature, line measurements were taken from the centroid of the juxtacentromeric heterochromatin signals to the extreme nuclear periphery or nuclear rim where the intensity value became less than one standard deviation above background. The microscope used for image capture had been previously calibrated with a stage-micrometer and a conversion factor of 0.135 µm pixel^−1^ was applied to images captured using the 100× objective. For the varying sizes of nuclei to be taken into account when measuring the distances of juxtacentromeric heterochromatin from the extreme nuclear periphery, the values were normalised between patient and control pairs. This was done by measuring the areas of DAPI stained nuclei by the Classifier feature in Volocity, using the percentage mode set to a lower limit of 12% intensity, an upper limit of 100% and excluding areas smaller than 5000 pixels. Tables of nuclear areas were exported for processing in Microsoft Excel, and the mean nuclear radius was calculated for each cell line. This allowed the ratios of distance from extreme nuclear periphery to nuclear radius to be calculated. The ratios for the ICF cell lines were 0.460 (Patient 1) and 0.512 (Patient 2). For the control cell lines, the ratios were 0.402 (Control 1), 0.364 (Control 2) and 0.337 (DO208915).

### Statistical analysis of FISH data

Categorical data, such as that obtained when making observations of association or non-association with the extreme nuclear periphery of juxtacentromeric heterochromatin or genes, were statistically analysed using a Chi-squared goodness of fit test. For these analyses, observed frequencies from the ICF samples were compared to expected frequencies, as obtained from the controls. Quantitative data from the area, volume and distance measurements, which demonstrate a normal distribution, were statistically analysed using the non-parametric Kolmogorov-Smirnov test. For Kolmogorov-Smirnov test: D = the maximum difference between the cumulative distributions; P = probability of the null hypothesis. A P value of ≤0.05 is generally accepted as having biological significance.

### Quantitative Real Time PCR

Total genomic DNA was prepared from each of the samples using Qiagen Blood and Cell Culture DNA Mini Kit as recommended by the manufacturer. For Real Time PCR, 50 ng of each individual DNA were amplified using the Sybr Green kit (Invitrogen) on an iCycler Bio-Rad (UK) Real-Time PCR system, using the following primers: hsSat2 (NCBI accession number X72623) 5′-ATCGAATGGAAATGAAAGGAGTCA-3′; 5′-GACCATTGGATGATTGCAGTCA-3′. S1/AS1 [64] 5′-AGTCCATTCAATGATTCCATTCCAGT-3′; 5′-AATCATCATCCAACGGAAGCTAATG-3′.

As a reference, primers for a single copy gene, *CENPB* (NCBI accession number NP 001801), 5′-GGCTTACTTTGCCATGGTCAA-3′ 5′-TTGATGTCCAAGACCTCGAACTC-3′ and Alu sequences (NCBI accession number D90162) were used.

5′-CTCCCGGATTCAAGCAATTA-3′

5′-CATGGTGAAACCCCATCTCT-3′

In each experiment, for each DNA sample, five replicates were run.

The Ct values were compared using the 2^−ΔΔCT^ formula.

### Gene expression analysis by Real-Time RT-PCR

Total RNA was extracted from B-lymphoblastoid cells from ICF patient 1 and 2, and Control 1 and 2 using the RNeasy extraction kit (Qiagen, UK) as recommended by the manufacturer. Reverse transcription reactions were performed to generate 2 µg of cDNA from 2 µg of total RNA. First strand synthesis was set up by adding 500 µg of Oligo(dT) (Invitrogen), 10 mM dATP, 10 mM dCTP, 10 mM dGTP and 10 mM dTTP to 2 µg of total RNA and incubating at 65°C for 5 minutes before chilling on ice for 5 minutes. 5× first stand buffer, 0.1 M DTT and 40 units of RNaseOUT™ (Invitrogen) were added to the reactions, which were incubated at 42°C for 2 minutes. 200 units of SuperScript™ II reverse transcriptase (Invitrogen) were added and the reactions incubated at 42°C for 50 minutes, before inactivation at 70°C for 15 minutes. The relative expression of *BTG2*, *CNN3*, *ID3*, *RGS1* and *F13A1* in ICF patient and control cell lines was analysed using the Bio-Rad iCycler system. Reactions were set-up with 100 ng of template cDNA, 500 nM primers and iQ SYBR Green PCR Supermix (Bio-Rad). β-actin was used as a normalisation gene. Primers suitable for real-time RT-PCR (Table S1) were designed with the LightCycler (Roche) primer design software using mRNA sequences of the genes obtained from the Human Genome Browser Gateway. Relative gene expression was calculated using the following formula [65]:
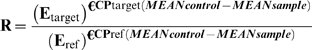



R = Relative expression ratio

E = Efficiency of PCR (calculated from E = 10^(−1/slope of optimisation curve)^)

MEAN = Normalisation gene

CP values were obtained from the Bio-Rad iCycler software when viewing post-run data

### Quantitative methylation analysis

Genomic DNA was extracted from B-lymphoblastoid cells from ICF patient 1 and 2, Control 1 and 2, and DO208915 using the GeneCatcher™ gDNA 3–10 mL Blood Kit (Invitrogen). 1 µg of genomic DNA was denatured and bisulfite treated using the EZ DNA Methylation-Gold Kit (Zymo Research, USA). Following this treatment, PCR was performed to amplify regions from genes *BTG2*, *CNN3* and *ID3*, using 10 µM tailed primers described in Table S2 and using Qiagen Hot Start Taq. PCR products were then processed using the MassCLEAVE kit reagents from Sequenome (San Diego, USA) to generate reverse strand specific fragments for MALDI_TOF mass spectrometry [27]. Conditioning of the phosphate backbone prior to MALDI-TOF mass spectrometry was performed by the addition of 6 mg of CLEAN resin (Sequenom, San Diego, USA). 15 nL of the cleavage reactions were robotically dispensed onto silicon chips preloaded with matrix (Sequenom, San Diego, USA). The mass spectra were obtained using the Autoflex MassARRAY mass spectrometer (Sequenom, San Diego, USA) and analysed using proprietary interpretation software tools.

## Supporting Information

Figure S1 FACS analysis. Cell cycle phase composition was analysed by FACS. The four unsynchronysed cell lines present similar percentages of diploid cells in G1, S and G2.
(0.09 MB TIF)

Figure S2 Effects of the 5-azacytidine on Control 1 cells. Metaphase spreads obtained from Control 1 after the demethylating treatment show variable extent of decondensation or stretching of the chromosome 1 juxtacentromeric heterochromatin (white arrows), similar to what normally observed in ICF cells. Panels C, D and E show dual colour FISH images with D1Z1 in green and D9Z3 in red. Chromosomes are counterstained with DAPI.
(2.08 MB TIF)

Figure S3 Effects of the 5-azacytidine on Control 2 cells. Similarly to what observed for Control 1, metaphase spreads obtained from Control 2 after the demethylating treatment show variable extent of decondensation or stretching of the chromosome 1 juxtacentromeric heterochromatin (white arrows), similar to what normally observed in ICF cells. Panels B and D show dual colour FISH images with D1Z1 in green and D9Z3 in red. Chromosome are counterstained with DAPI.
(1.78 MB TIF)

Figure S4 Quantitative methylation analysis of *BTG2*, *CNN3* and *ID3*. The methylation status of promoter CpG islands upstream of genes *BTG2*, *CNN3* and *ID3* was investigated using a MALDI-TOF based quantitative methylation assay on bisulphite treated DNA (for details see Materials and Methods). The EpiGrams summarise the data from the C and T specific cleavage reactions (internal and external circle respectively), the detected methylation level is represented as colour gradient based on the percentage of methylation detected by the analysis. The specific CpG sites for each gene CpG island analysed are numbered and shown in the specific base pair position within the specific amplicon: *BTG2* (A), *CNN3* (B) and *ID3* (C). ICF Patients 1 and 2, Controls 1 and 2 and additional controls of DO208915, methylated control DNA (Chemicon, USA), hemi-methylated control DNA obtained mixing an unmethylated and a methylated control in equal concentration.
(5.43 MB TIF)

Figure S5 Intra-nuclear measurements of the juxtacentromeric heterochromatic areas on 2D fixed cells. The nuclear areas occupied by the juxtacentromeric heterochromatic regions, as defined by hybridisation on 2D-fixed interphase nuclei with the corresponding classical satellite DNA probes, were measured. Raw images were thresholded using the Classifier feature of Volocity at a level which excluded background fluorescence with the threshold set to a lower limit of 27% intensity, and an upper limit of 100%. Both limits were defined empirically. Areas smaller than 25 pixels and larger than 500 pixels were excluded. The resulting areas, outlined in the images by a dashed line, were measured and exported as data tables for analysis in Excel. Examples of different hybridisation patterns: conventional (A) versus compact (B).
(1.14 MB TIF)

Table S1 Primer pairs used for PCR and real-time RT-PCR. Primers used for PCR to validate the presence of the correct insert in the BAC clones were generated from genomic sequences obtained from the Human Genome Browser Gateway and detailed in the table above. Primers used for quantitative real-time reverse-transcription PCR (RT-PCR) were generated from mRNA sequences of the genes of interest obtained from the Human Genome Browser Gateway and are also detailed above.
(0.08 MB JPG)

Table S2 Primers for PCR reactions prior to quantitative methylation analysis using the Sequenom mass spectrometer. The sequences of the primers used to amplify the promoter CpG islands of genes *BTG2*, *CNN3* and *ID3* and the sizes of products expected from the PCR reactions.
(0.04 MB JPG)
