# Supplementary figures and images for: Altered Intra-Nuclear Organisation of Heterochromatin and Genes in ICF Syndrome
Source: PLoS One. 2010 Jun 29;5(6):e11364. doi: 10.1371/journal.pone.0011364 (PMC2894064; doi:10.1371/journal.pone.0011364)

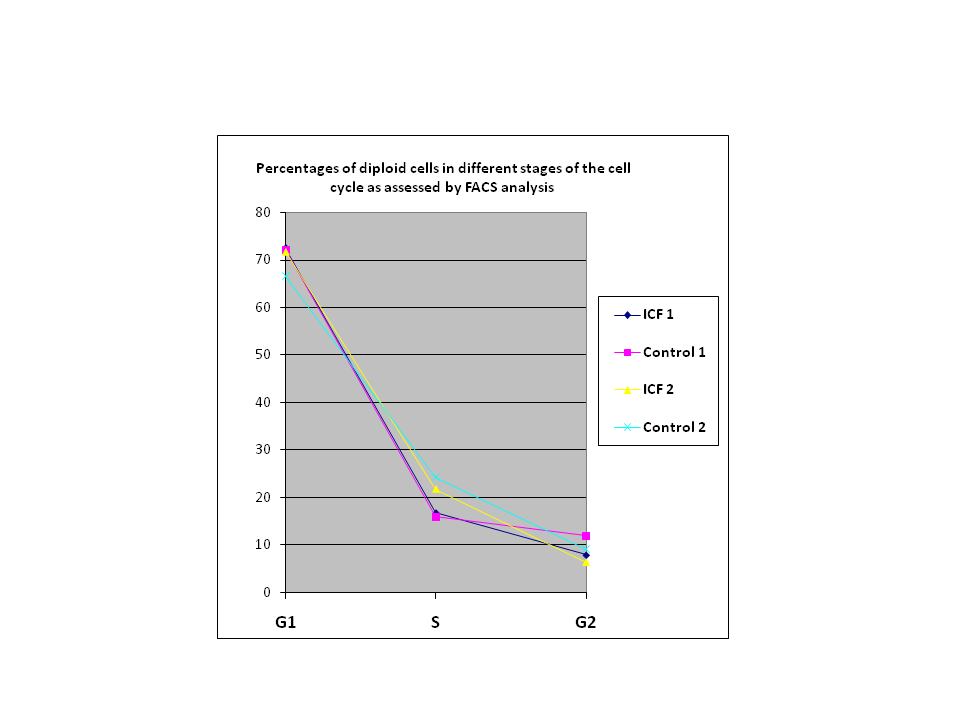

Supplement: Figure S1 — FACS analysis. Cell cycle phase composition was analysed by FACS. The four unsynchronysed cell lines present similar percentages of diploid cells in G1, S and G2. (0.09 MB TIF) [file pone.0011364.s001.tif]

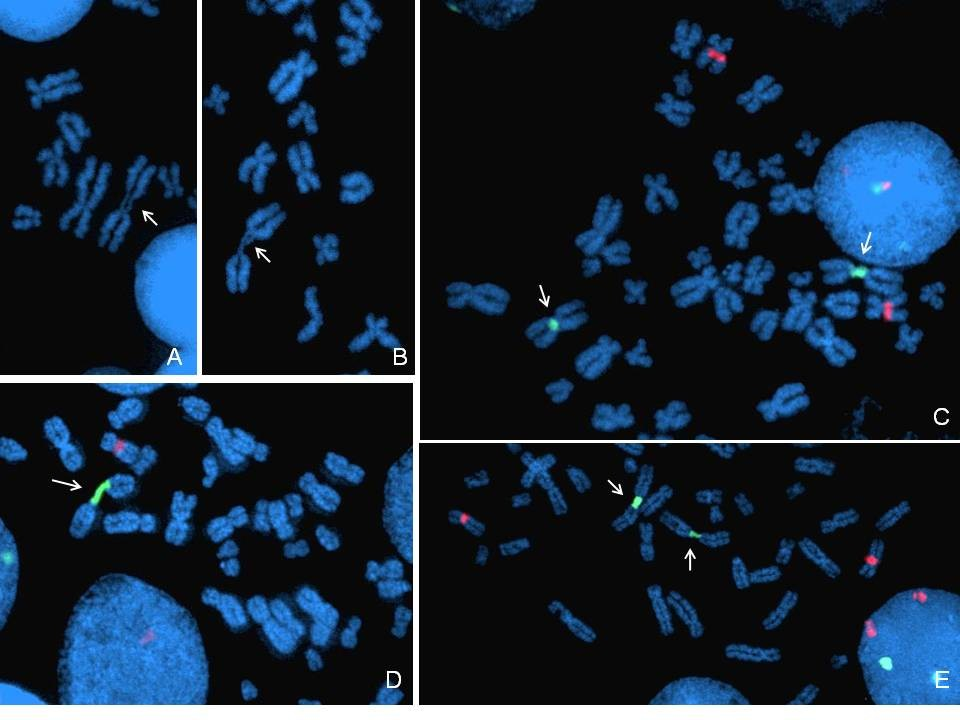

Supplement: Figure S2 — Effects of the 5-azacytidine on Control 1 cells. Metaphase spreads obtained from Control 1 after the demethylating treatment show variable extent of decondensation or stretching of the chromosome 1 juxtacentromeric heterochromatin (white arrows), similar to what normally observed in ICF cells. Panels C, D and E show dual colour FISH images with D1Z1 in green and D9Z3 in red. Chromosomes are counterstained with DAPI. (2.08 MB TIF) [file pone.0011364.s002.tif]

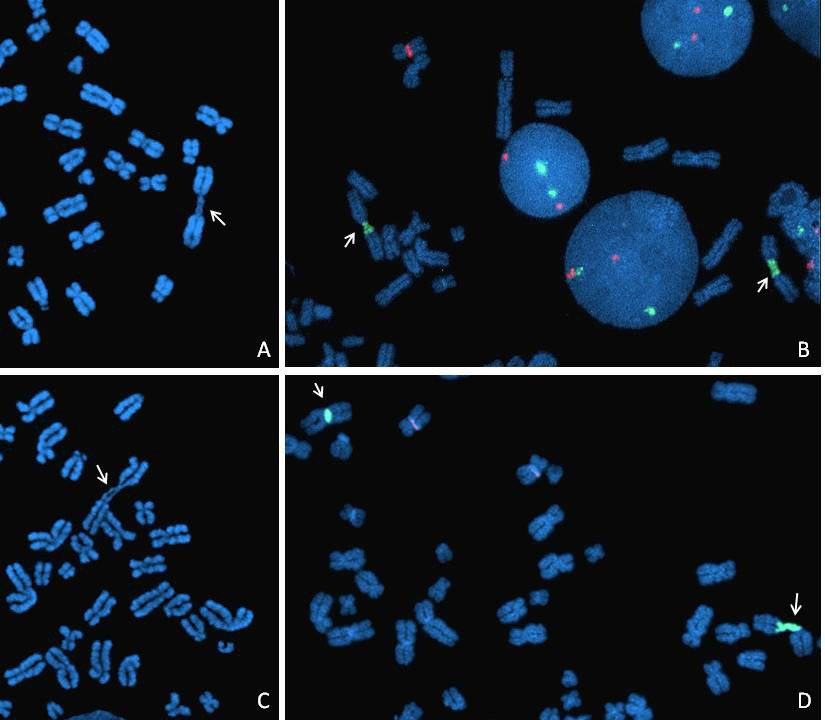

Supplement: Figure S3 — Effects of the 5-azacytidine on Control 2 cells. Similarly to what observed for Control 1, metaphase spreads obtained from Control 2 after the demethylating treatment show variable extent of decondensation or stretching of the chromosome 1 juxtacentromeric heterochromatin (white arrows), similar to what normally observed in ICF cells. Panels B and D show dual colour FISH images with D1Z1 in green and D9Z3 in red. Chromosome are counterstained with DAPI. (1.78 MB TIF) [file pone.0011364.s003.tif]

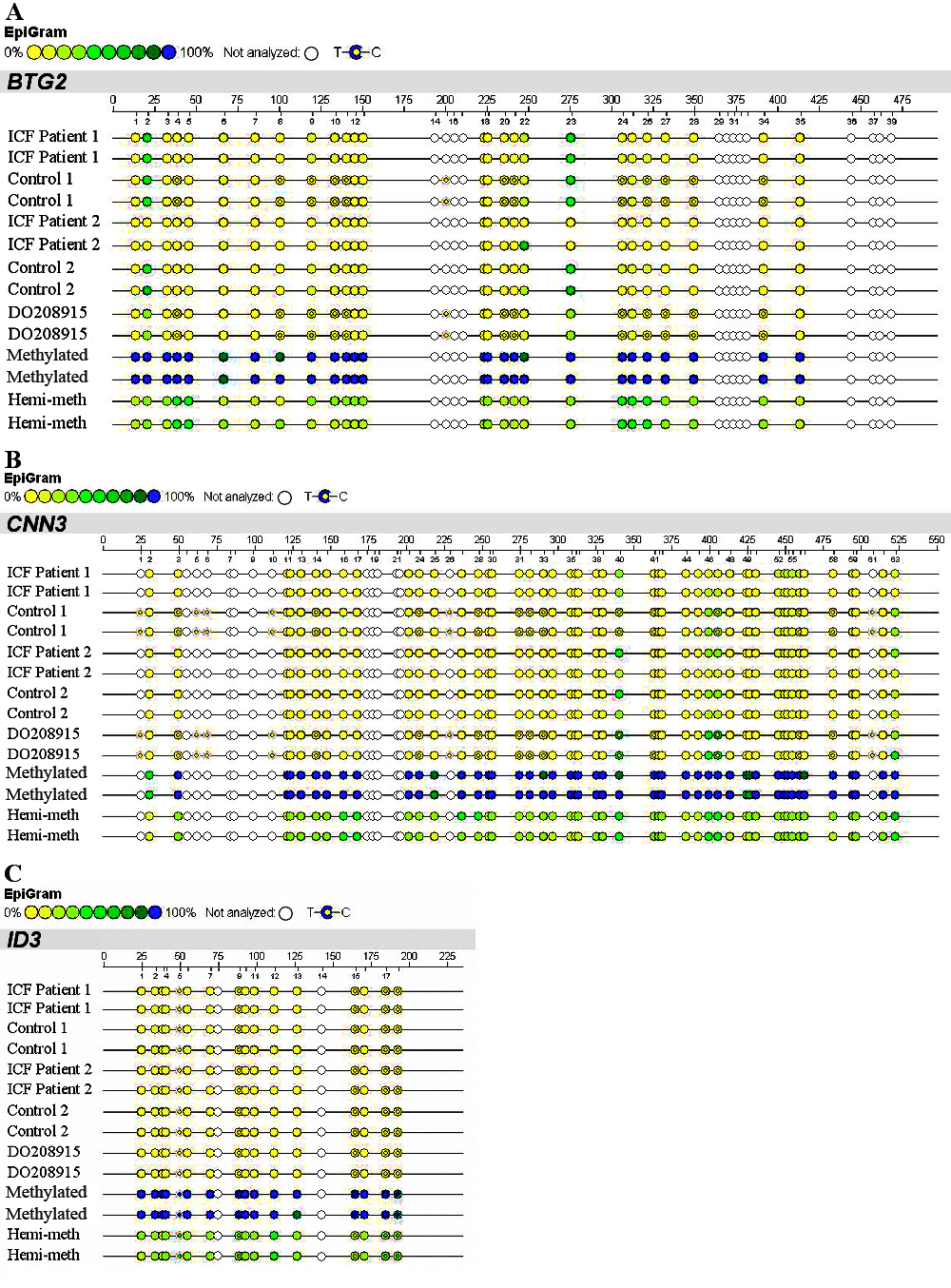

Supplement: Figure S4 — Quantitative methylation analysis of BTG2, CNN3 and ID3. The methylation status of promoter CpG islands upstream of genes BTG2, CNN3 and ID3 was investigated using a MALDI-TOF based quantitative methylation assay on bisulphite treated DNA (for details see Materials and Methods). The EpiGrams summarise the data from the C and T specific cleavage reactions (internal and external circle respectively), the detected methylation level is represented as colour gradient based on the percentage of methylation detected by the analysis. The specific CpG sites for each gene CpG island analysed are numbered and shown in the specific base pair position within the specific amplicon: BTG2 (A), CNN3 (B) and ID3 (C). ICF Patients 1 and 2, Controls 1 and 2 and additional controls of DO208915, methylated control DNA (Chemicon, USA), hemi-methylated control DNA obtained mixing an unmethylated and a methylated control in equal concentration. (5.43 MB TIF) [file pone.0011364.s004.tif]

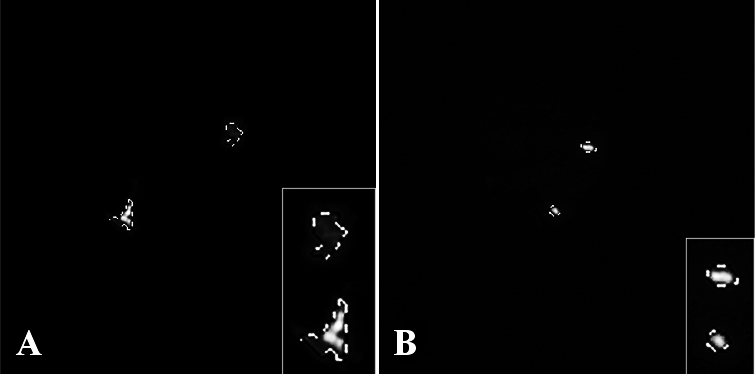

Supplement: Figure S5 — Intra-nuclear measurements of the juxtacentromeric heterochromatic areas on 2D fixed cells. The nuclear areas occupied by the juxtacentromeric heterochromatic regions, as defined by hybridisation on 2D-fixed interphase nuclei with the corresponding classical satellite DNA probes, were measured. Raw images were thresholded using the Classifier feature of Volocity at a level which excluded background fluorescence with the threshold set to a lower limit of 27% intensity, and an upper limit of 100%. Both limits were defined empirically. Areas smaller than 25 pixels and larger than 500 pixels were excluded. The resulting areas, outlined in the images by a dashed line, were measured and exported as data tables for analysis in Excel. Examples of different hybridisation patterns: conventional (A) versus compact (B). (1.14 MB TIF) [file pone.0011364.s005.tif]

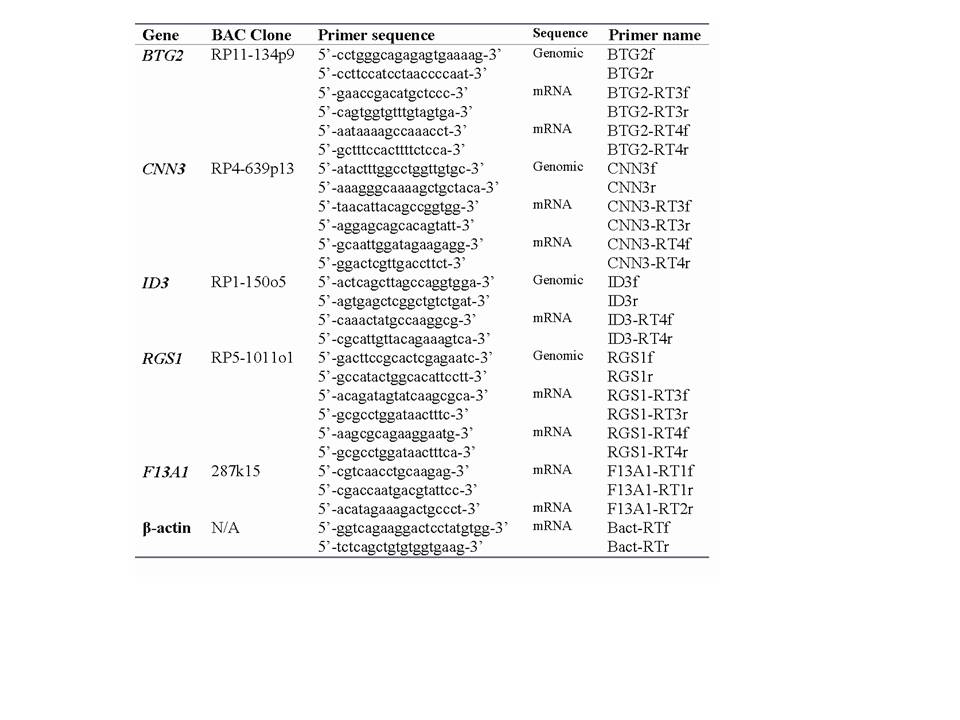

Supplement: Table S1 — Primer pairs used for PCR and real-time RT-PCR. Primers used for PCR to validate the presence of the correct insert in the BAC clones were generated from genomic sequences obtained from the Human Genome Browser Gateway and detailed in the table above. Primers used for quantitative real-time reverse-transcription PCR (RT-PCR) were generated from mRNA sequences of the genes of interest obtained from the Human Genome Browser Gateway and are also detailed above. (0.08 MB JPG) [file pone.0011364.s006.jpg]

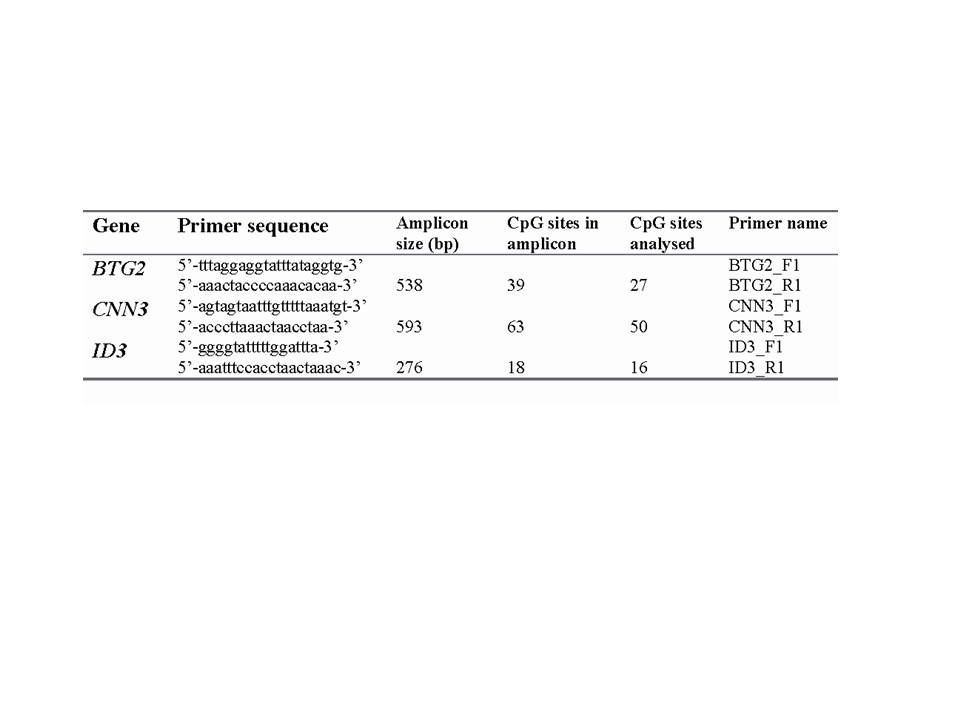

Supplement: Table S2 — Primers for PCR reactions prior to quantitative methylation analysis using the Sequenom mass spectrometer. The sequences of the primers used to amplify the promoter CpG islands of genes BTG2, CNN3 and ID3 and the sizes of products expected from the PCR reactions. (0.04 MB JPG) [file pone.0011364.s007.jpg]
